# Prediction of allergic disease trajectories from birth up to adolescence

**DOI:** 10.1111/pai.70341

**Published:** 2026-04-12

**Authors:** Miriam Leskien, Martin Scheerer, Elisabeth Thiering, Sara Kress, Claire Coffey, Dietrich Berdel, Andrea von Berg, Carl‐Peter Bauer, Monika Gappa, Joachim Heinrich, Sibylle Koletzko, Tamara Schikowski, Berthold Koletzko, Annette Peters, Marie Standl

**Affiliations:** ^1^ Institute of Epidemiology Helmholtz Zentrum München‐German Research Center for Environmental Health Neuherberg Germany; ^2^ Institute for Medical Information Processing, Biometry, and Epidemiology LMU Munich Munich Germany; ^3^ German Center for Child and Adolescent Health (DZKJ) Munich Germany; ^4^ Department of Paediatrics Dr. von Hauner Children's Hospital, LMU University Hospital Munich Germany; ^5^ IUF‐Leibniz Research Institute for Environmental Medicine Düsseldorf Germany; ^6^ Research Institute, Department of Pediatrics Marien‐Hospital Wesel Wesel Germany; ^7^ Department of Pediatrics Technical University of Munich Munich Germany; ^8^ Evangelisches Krankenhaus Düsseldorf Children's Hospital Düsseldorf Germany; ^9^ Institute and Clinic for Occupational, Social and Environmental Medicine University Hospital, LMU Munich Munich Germany; ^10^ Allergy and Lung Health Unit, Melbourne School of Population and Global Health The University of Melbourne Melbourne Victoria Australia; ^11^ German Center for Lung Research (DZL) Munich Germany; ^12^ Department of Pediatrics, Gastroenterology and Nutrition, School of Medicine Collegium Medicum University of Warmia and Mazury Olsztyn Poland; ^13^ Chair of Epidemiology Ludwig‐Maximilians‐Universität München Munich Germany

**Keywords:** allergic disease trajectories, allergic multimorbidity, early life, machine learning, prediction

## Abstract

**Background:**

Allergic diseases often develop jointly during early childhood. Potential disease trajectories and relevant early‐life factors have been described, yet existing prediction approaches mostly focus on single allergic diseases cross‐sectionally. Models addressing allergic multimorbidity and disease trajectories are lacking. We aim to predict allergic disease trajectories from birth up to adolescence using early‐life factors.

**Methods:**

Preceding research using data from 4646 adolescents of the German birth cohorts GINIplus and LISA identified seven allergic disease trajectories up to the age of 15 years. A set of predictors comprising parental and perinatal factors, early allergic or respiratory symptoms, lifestyle and environmental factors was used with an XGBoost machine learning approach to perform multiclass classification. In a subsample (*N* = 2109), polygenic risk scores (PRS) for asthma, allergic rhinitis, atopic dermatitis, and any allergy were added to the predictor set.

**Results:**

Our approach revealed moderate classification success (multiclass area under the curve (AUC) = 0.69). A macro‐averaged sensitivity of 0.26 and specificity of 0.89 were obtained. The most important predictors were early‐life skin rash, respiratory symptoms, and air pollution. In the sub‐analysis, the PRS were among the factors with high importance, but the prediction performance in external test data was not improved.

**Conclusions:**

Our prediction success was comparable to established prediction scores while accounting for multiple allergic disease trajectories and using solely early‐life factors. This study cannot yet provide reliable individual‐level prediction in a clinical setting but can inform development of future work on this.

**Abbreviations**
API: Asthma Predictive Index
ASPIRE: ASthma PredIctive Risk scorE
AUC: Area Under the Curve
BMI: Body Mass Index
GINIplus: German Infant Nutritional Intervention plus environmental and genetic influences on allergy development
lasso: Least Absolute Shrinkage and Selection Operator
LISA: Influence of Life‐style factors on the development of the Immune System and Allergies
PARS: Pediatric Asthma Risk Score
PRS: Polygenic Risk Score
ROC: Receiver Operating Characteristic
SHAP: SHapley Additive exPlanations

**Key message**

Predicting trajectories of allergic diseases from birth to adolescence yields a performance comparable to existing prediction models–limited to the prediction of single allergic disease entities at specific time points–while accounting for the complex interplay among the three allergic diseases asthma, allergic rhinitis, and atopic dermatitis. Using a machine learning approach and easily accessible early life factors as predictors, early life determinants most relevant to distinct allergic disease trajectories and their differentiation were identified. Although the performance is moderate and therefore limits reliable individual‐level prediction, this study can inform future work on the development of such models.

## 1 INTRODUCTION

Allergic diseases present a major public health burden, affecting approximately 10% to 30% of the global population^1^, and there is no cure available. Asthma, allergic rhinitis and atopic dermatitis are among the most common allergic conditions with a particularly high prevalence in childhood and adolescence.^2^ They often coexist, known as allergic multimorbidity, and are suggested to have shared molecular pathways. The evident rise in the prevalence of allergic diseases over recent decades^3^ highlights the role of environmental and lifestyle factors in their development.^4^ For instance, air pollution^5^ or environmental tobacco smoke exposure^6^ have been associated with an increased risk for the development and progression of allergic diseases. Due to the critical window of immune maturation in the first years of life, perinatal and early‐life environmental and lifestyle factors are particularly relevant.^7^ The heterogeneity of allergic disease phenotypes and the fact that some children outgrow symptoms while others experience persistent or worsening conditions into adolescence and adulthood implies diverse trajectories of disease. Such trajectories have been identified by several studies.^8^, ^9^, ^10^ Differential genetic associations with allergic disease developmental profiles also suggest distinct biological mechanisms.^11^ For targeted prevention, it would be beneficial to predict these different allergic disease trajectories in early life to identify children with high risk of disease progression later in life. Several attempts have already been made to develop an easily‐applicable asthma prediction score: The Asthma Predictive Index (API) which is often used in clinical practice but criticized for its low sensitivity,^12^ the Pediatric Asthma Risk Score (PARS) which improves the prediction of mild‐to‐moderate asthma risk,^13^ and the ASthma PredIctive Risk scorE (ASPIRE) which also considers asthma development and persistence up to young adult age.^14^ Although the latter two yield better sensitivity compared to the API, they focus on the prediction of asthma alone, predominantly using information on early‐life symptoms but mostly neglecting early‐life environmental and lifestyle factors that are known to be relevant for asthma risk later in life. More complex supervised machine learning approaches do consider some of these early‐life determinants and further improve performance.^15^, ^16^ However, they are also restricted to the prediction of asthma at a certain age without considering other allergic diseases or time points. Only very few studies predict allergic rhinitis or atopic dermatitis based on early‐life factors using machine learning^17^, ^18^ but do not report the performance of their model in an external dataset. However, no prediction model considering the complex interplay of different allergic diseases from birth until adolescence has been reported. Therefore, we aim to implement and externally validate a machine learning approach to predict allergic disease trajectories^8^ from birth until adolescence on a population level based on easily accessible early‐life factors and to identify relevant modifiable early‐life factors as potential targets for prevention strategies.

## 2 METHODS

### 2.1 Study population

Data from the prospective population‐based German birth cohorts GINIplus (German Infant Nutritional Intervention plus environmental and genetic influences on allergy development) and LISA (Influence of Life‐style factors on the development of the Immune System and Allergies in East and West Germany) were used. Full‐term, healthy newborns were recruited in Munich and Wesel between 1995 and 1998 for GINIplus, and in Munich, Wesel, Bad Honnef and Leipzig between 1997 and 1999 for LISA. Sufficient knowledge of the German language was an inclusion criterion for both studies, and the study population was of mostly European ancestry. Ethical approval for both studies was obtained from local ethics committees and all participants or their caregivers gave written informed consent. Further information is provided by Heinrich et al.^19^ This study uses data from the Munich and Wesel study center with air pollution data available. In total, it includes data from 4646 children from birth until adolescence, using GINIplus (*N* = 3277) for model training and LISA (*N* = 1369) for external validation. Missing values of early life determinants were single‐imputed using Multivariate Imputation by Chained Equations (MICE) with the *mice* package^20^ (for details see Supplemental Methods).

### 2.2 Allergic disease trajectories

Previous work derived allergic disease trajectories in the GINIplus and LISA studies reflecting different patterns of onset, persistence, multimorbidity and remission from birth until the age of 15 years, considering yearly parent‐reported asthma, atopic dermatitis and allergic rhinitis doctor diagnoses.^8^ Using longitudinal k‐means clustering, the following trajectories were identified: “Intermittently allergic” (17.5%) with mild or transient allergic disease, “Rhinitis” (7.5%) with allergic rhinitis but very low asthma and atopic dermatitis prevalence, “Early‐resolving dermatitis” (6.3%) including children with atopic dermatitis within the first years of life and mostly experiencing remission, “Mid‐persisting dermatitis” (4.1%) with children developing dermatitis mainly during mid‐childhood up to the age of 10 years and more than 50% persistence at age 15, “Multimorbid” (4.0%) with almost all children developing asthma combined with a relatively high prevalence of rhinitis and dermatitis, “Persisting dermatitis plus rhinitis” (2.2%) where most children had atopic dermatitis early in life and then develop rhinitis with a high proportion of persisting dermatitis, and “Early‐transient asthma” (0.5%) characterized by early wheezing and subsequent remission. The early‐transient asthma trajectory was excluded for this study due to small sample size (*N* = 27). Children never diagnosed with any allergic disease were assigned to a non‐allergic trajectory beforehand.^8^

### 2.3 Selection of early‐life predictor set

Fifty‐six candidates consisting of parental, perinatal, environmental, and lifestyle factors as well as early allergic or respiratory symptoms within the first 4 years of life were selected based on existing literature (Table S1).

### 2.4 Model training and optimization

**FIGURE 1:**
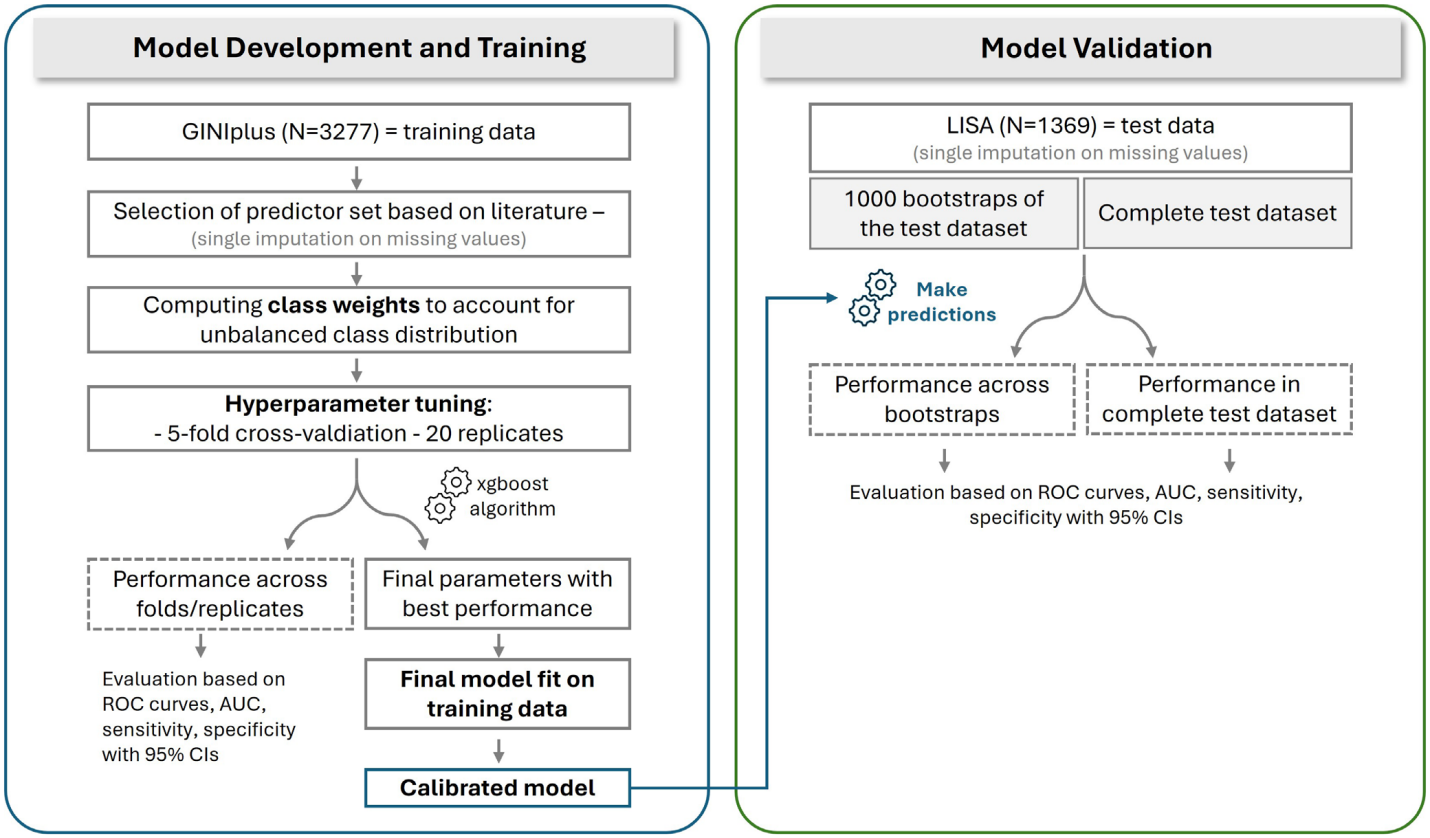
Workflow for the model development and training (left blue part) and external validation of the model (right green part).

Allergic disease trajectories including the non‐allergic trajectory as reference were predicted via multiclass classification using the set of selected early‐life factors. We applied XGBoost, a supervised decision tree‐based machine learning approach, which has been shown to perform well in multi‐class imbalanced data,^21^ using the *xgboost* ^22^ V1.7.8.1 package. Model training was performed in training data (GINIplus) and followed by external validation of the model in the independent test dataset (LISA) (Figure 1). To account for the unbalanced class distribution, class weights were used to give more importance to underrepresented classes during model training. To find the optimal hyperparameters for the XGBoost algorithm, parameter tuning was performed in the training data by the *tune* V1.2.1 package. This was done via grid search and cluster‐stratified 5‐fold cross‐validation which preserved the overall distribution of allergic disease trajectories, iterating over 20 replicates. For cross validation, the *glmnet* ^23^ V4.1.8 package was used. In this step, the combination of model settings that yields the best predictive performance was searched for by systematically testing numerous combinations of parameters in the training data, using four parts of it for training and the fifth for testing the fitted model based on these parameters. The best identified parameters were used to train the final model on the training dataset. The final model was then calibrated by using a multinomial logistic regression predicting the true trajectories based on the predicted probabilities in the training data. Calibration was assessed using calibration plots with flexible Locally Estimated Scatterplot Smoothing (LOESS) curves, by applying the *CalibrationCurves* ^24^, ^25^, ^26^, ^27^ V3.0.0 package, and the multiclass brier score on the original scale (range 0–2).^28^

### 2.5 External validation

The final calibrated model was used to make predictions in the test dataset for external validation. Additionally, it was tested in 1000 bootstraps of the test dataset to generate 95% confidence intervals, derived by the *rsample* ^29^ V1.3.0 package.

### 2.6 Assessment of performance

To assess the classification performance, receiver operating characteristic (ROC) curves indicating the model's ability to discriminate between membership in a given trajectory versus all other trajectories were created and AUC values per allergic disease trajectory were computed by one‐versus‐all classification based on calculated probabilities for each class. Sensitivity, specificity, positive and negative likelihood ratio were calculated for each trajectory. Additionally, the macro‐averaged multiclass AUC,^30^ sensitivity and specificity were calculated. For computing classification performance metrics the *MLmetrics* ^31^ V1.1.3 package was used. For ROC curves, AUC, sensitivity and specificity, 95% confidence intervals were derived. For internal validation, these were derived from the performance across cross‐validation folds and replicates; for external validation, bootstrap iterations were used. These were reported along with mean performance metrics.

Additionally, to find the best balance between sensitivity and specificity, the Closest Point method was used. To assess clinical utility, a decision curve analysis was performed for each trajectory individually using a one‐versus‐all approach and computing the net benefit of the prediction of the respective trajectory.

### 2.7 Variable importance and contribution of single predictors

The variable importance was derived from the model using the *vip* ^32^ V0.4.1 package to identify the factors most valuable for making predictions (for details see Supplemental Methods). SHapley Additive exPlanations (SHAP)^33^ were calculated by the *xgboost* ^22^ V1.7.8.1 package and used to evaluate the contributions of the single features on the prediction of each trajectory.

### 2.8 Sub‐analysis using genetic data

In a subsample (*N* = 2109) of European ancestry with genetic data available, polygenic risk scores (PRS) for asthma,^34^ allergic rhinitis,^35^ atopic dermatitis,^36^ and any allergy^37^ were added to the predictor set to evaluate whether the inclusion of PRS could improve predictions. The performance in training (GINIplus, *N* = 1358) and test (LISA, *N* = 751) data was compared within the subsample with or without adding PRS using the same methods as before. Details on the PRS construction are described in the Supplemental Methods. All analyses were performed in R^38^ V4.4.1.

## 3 RESULTS

### 3.1 Study population

In total, 3277 participants from GINIplus were included in the training and 1369 participants from LISA in the test dataset, with similar sex distribution (Table S2). The assignment to allergic disease trajectories based on GINIplus and LISA by Kilanowski et al.^8^ is shown in Table 1.

**TABLE 1:** Frequencies of allergic disease trajectories.

	Training dataset (GINIplus)	Test dataset (LISA)	Overall
	(*N* = 3277)	(*N* = 1369)	(*N* = 4646)
No allergy	1831 (55.9%)	869 (63.5%)	2700 (58.1%)
Intermittently allergic	612 (18.7%)	214 (15.6%)	826 (17.8%)
Rhinitis	263 (8.0%)	89 (6.5%)	352 (7.6%)
Early‐resolving dermatitis	213 (6.5%)	75 (5.5%)	288 (6.2%)
Mid‐persisting dermatitis	136 (4.2%)	50 (3.7%)	186 (4.0%)
Multimorbid	139 (4.2%)	45 (3.3%)	184 (4.0%)
Persisting dermatitis + rhinitis	83 (2.5%)	27 (2.0%)	110 (2.4%)

The predictor set consists of parental, perinatal and early‐life (within the first 4 years of life) determinants listed in Table S1. It comprises sex, parental education, family history of allergies, birth mode and weight, breastfeeding, maternal smoking and smoke exposure during pregnancy, presence of older siblings, BMI, indoor smoking at home, use of gas for cooking, mold, traffic‐related air pollution, urbanicity, contact with other children or daycare, cat and dog ownership as well as allergic or other respiratory symptoms including skin rash, wheezing, having an itchy or blocked nose without presence of a cold, airway infections, dry coughing at night without presence of a cold/bronchitis and having itchy/watery eyes together with nasal symptoms. While the training and test datasets were similar regarding the distribution of most of these variables, allergic symptoms like skin rash or wheezing were more prevalent in the LISA test dataset compared to the GINIplus training dataset (Table S2). In addition, in the LISA test dataset, more participants were from the urban Munich study center than rural Wesel compared to the training dataset participants.

### 3.2 Classification performance

**FIGURE 2:**
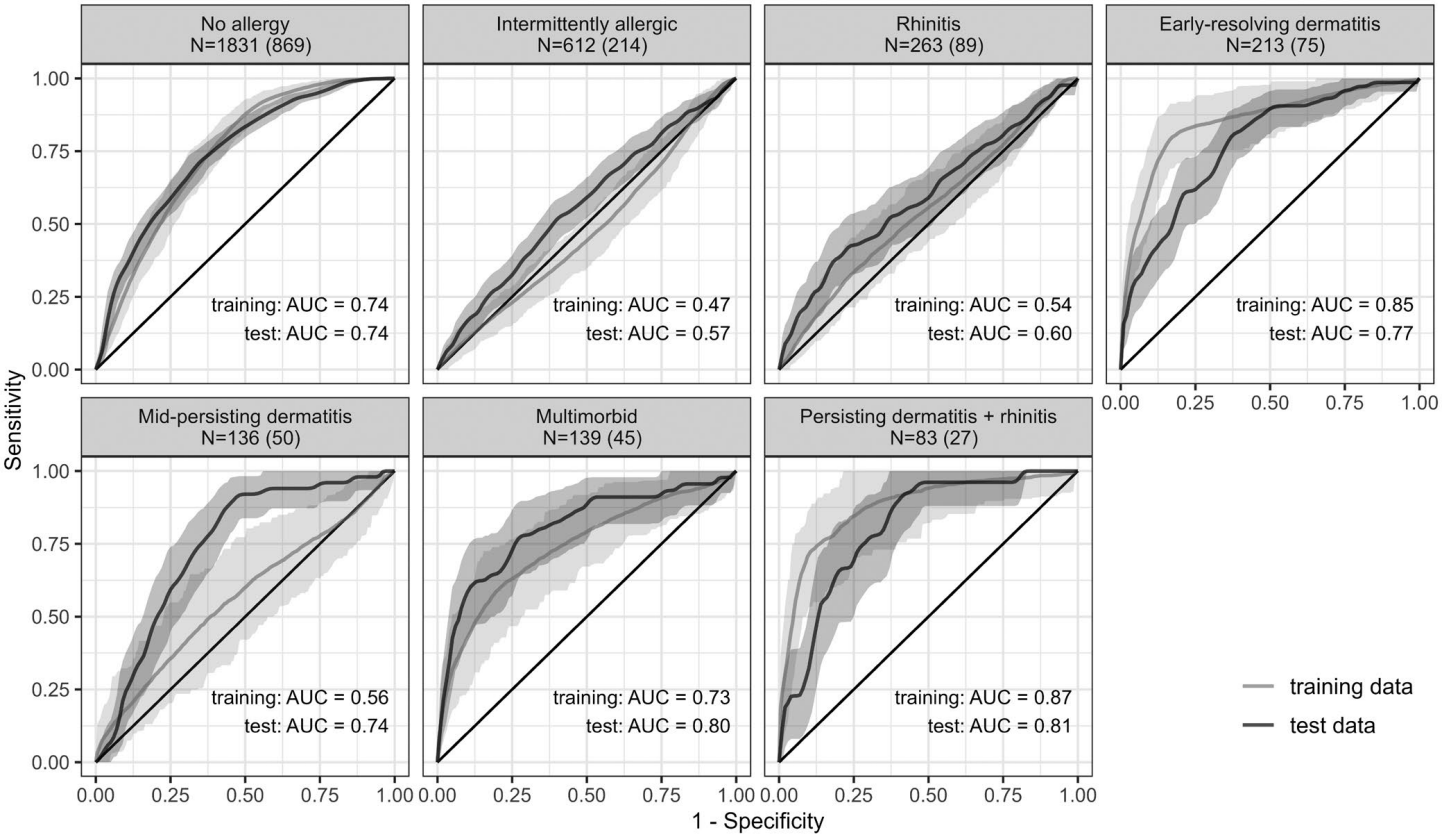
Receiver operator characteristic (ROC) curves per trajectory in training and test dataset, shown as mean performance complemented by 95% confidence intervals derived from performances across iterations over cross‐validation folds and replicates for training and bootstraps for test data. Sample size per trajectory is shown for the training dataset followed by the values for the test dataset in brackets. Mean AUC values are presented for training and test data.

Within the training data a multiclass AUC of 0.80, sensitivity of 0.44 and specificity of 0.90 were reached. In external validation, the model revealed moderate classification success with a multiclass AUC of 0.69, when using the complete test set. A macro‐averaged sensitivity of 0.26 and specificity of 0.89 was obtained. Trajectory‐specific performance metrics, including AUC, sensitivity and specificity, are presented in Table 2 along with 95% confidence intervals. ROC curves indicating the model's ability to discriminate between membership in a given trajectory versus all other trajectories are shown in Figure 2 along with AUC values. The curves represent the mean performance across folds and replicates for training data, and bootstraps of test data, and are presented along with 95% confidence intervals. For external validation in the complete test dataset, ROC curves are shown in Figure S1 and performance metrics in Table 2. The sensitivity of 0.72 in the non‐allergic trajectory indicates that the model correctly identifies a moderate proportion of true non‐allergic cases. However, the specificity of 0.64 implies that some individuals were misclassified as non‐allergic. The low sensitivity for almost all allergic trajectories indicates that the model fails to capture all individuals belonging to a certain trajectory. The specificity is high for all allergic disease trajectories indicating that most negative cases are correctly identified. The best prediction success was obtained for the early‐resolving dermatitis, multimorbid and persisting dermatitis + rhinitis trajectory with AUC values of 0.77, 0.80 and 0.81, and positive likelihood ratio values between 4.11 up to 6.50 (Table S3). The positive likelihood ratio represents the factor by which the odds of belonging to a certain trajectory increase when being assigned to the respective trajectory; the negative likelihood ratio the factor by which the odds of belonging to a certain trajectory decrease when not being assigned to the respective trajectory.

**TABLE 2:** Prediction performance metrics in training data from repeated cross‐validation and bootstrapped as well as complete test data: AUC, sensitivity and specificity.

Trajectory	Training performance (Internal validation)			Test performance (External validation)					
	Cross‐validation with 5 folds and 20 replicates (using training data)			Bootstrapped test datasets (*N* = 1000)			Complete test dataset		
	AUC	Sensitivity	Specificity	AUC	Sensitivity	Specificity	AUC	Sensitivity	Specificity
	Mean [95% CI]			Mean [95% CI]					
No allergy	0.74 [0.74; 0.74]	0.70 [0.69; 0.70]	0.64 [0.63; 0.64]	0.74 [0.72; 0.77]	0.74 [0.71; 0.77]	0.61 [0.57; 0.65]	0.74	0.72	0.64
Intermittently allergic	0.47 [0.47; 0.47]	0.03 [0.02; 0.03]	0.98 [0.98; 0.98]	0.57 [0.52; 0.61]	0.22 [0.16; 0.27]	0.87 [0.85; 0.89]	0.56	0.27	0.85
Rhinitis	0.54 [0.53; 0.54]	0.16 [0.16; 0.17]	0.89 [0.89; 0.89]	0.60 [0.53; 0.66]	0.11 [0.05; 0.18]	0.97 [0.96; 0.98]	0.60	0.10	0.97
Early‐resolving dermatitis	0.85 [0.84; 0.85]	0.52 [0.51; 0.53]	0.92 [0.91; 0.92]	0.77 [0.71; 0.82]	0.37 [0.26; 0.48]	0.91 [0.89; 0.92]	0.77	0.37	0.91
Mid‐persisting dermatitis	0.56 [0.55; 0.56]	0.11 [0.11; 0.12]	0.96 [0.96; 0.96]	0.74 [0.68; 0.80]	0.06 [0.00; 0.13]	0.96 [0.95; 0.97]	0.74	0.06	0.97
Multimorbid	0.73 [0.72; 0.73]	0.32 [0.31; 0.33]	0.92 [0.92; 0.92]	0.80 [0.72; 0.88]	0.14 [0.04; 0.25]	0.98 [0.98; 0.99]	0.80	0.11	0.98
Persisting dermatitis + rhinitis	0.87 [0.86; 0.87]	0.43 [0.42; 0.44]	0.95 [0.95; 0.95]	0.81 [0.73; 0.87]	0.23 [0.08; 0.39]	0.96 [0.95; 0.97]	0.81	0.22	0.96

**FIGURE 3:**
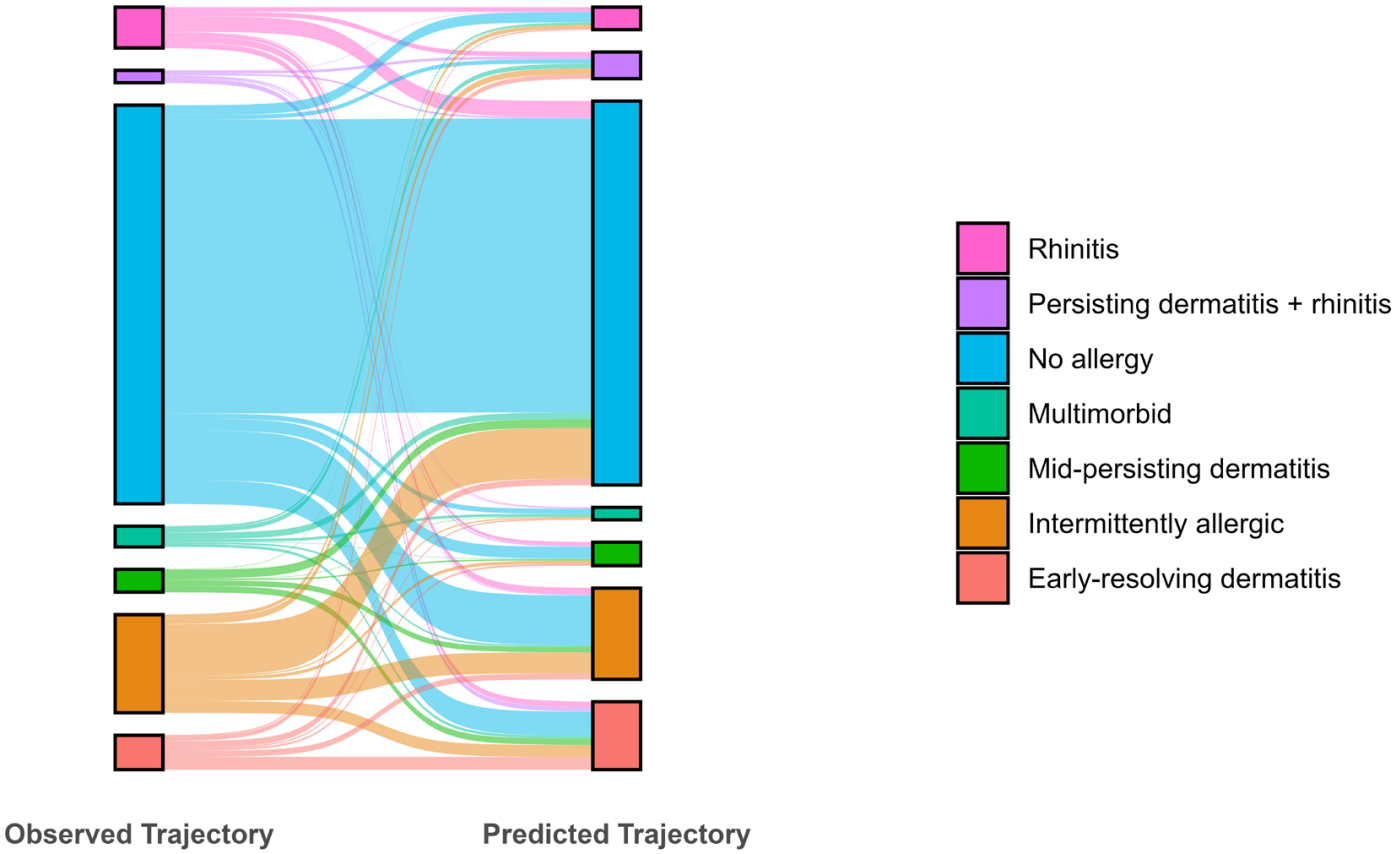
Sankey Plot illustrating the distributions of observed and predicted trajectories and their overlaps. Detailed numbers can be found in Table S4.

Additionally, the prediction performance is illustrated by the distribution of predicted versus observed trajectories (Figure 3, Table S4): in all predicted allergic trajectory groups, the highest number of misclassified individuals comes from the non‐allergic trajectory. Among the individuals misclassified as non‐allergic, the largest proportion belongs to the intermittently allergic trajectory. Another part of the non‐allergic trajectory is incorrectly classified into the early‐resolving dermatitis trajectory. Most of the intermittently allergic children are either predicted as such, as non‐allergic, or as part of the early‐resolving dermatitis trajectory.

Model calibration expressed by calibration plots (Figures S2 and S3) and a multiclass brier score of 0.84 before and 0.59 after recalibration (on a scale ranging 0–2) indicate that the initial poor calibration was improved after the recalibration procedure to better reflect true class distributions. Nevertheless, some classes, particularly the non‐allergic trajectory, still show systematic underprediction with points lying above the 45° identity line, while others show systematic overprediction with points well below the identity line. So even after recalibration, the plots do not clearly demonstrate good calibration.

Additionally, the probability thresholds with the best balance between sensitivity and specificity based on the Closest Point method are presented in Table S5 resulting in better sensitivity values at the expense of specificity.

A decision curve analysis revealed that the clinical utility of a prediction – specifically of one of the allergic disease trajectories – remains limited (Figure S4).

### 3.3 Variable importance and contribution of single predictors

**FIGURE 4:**
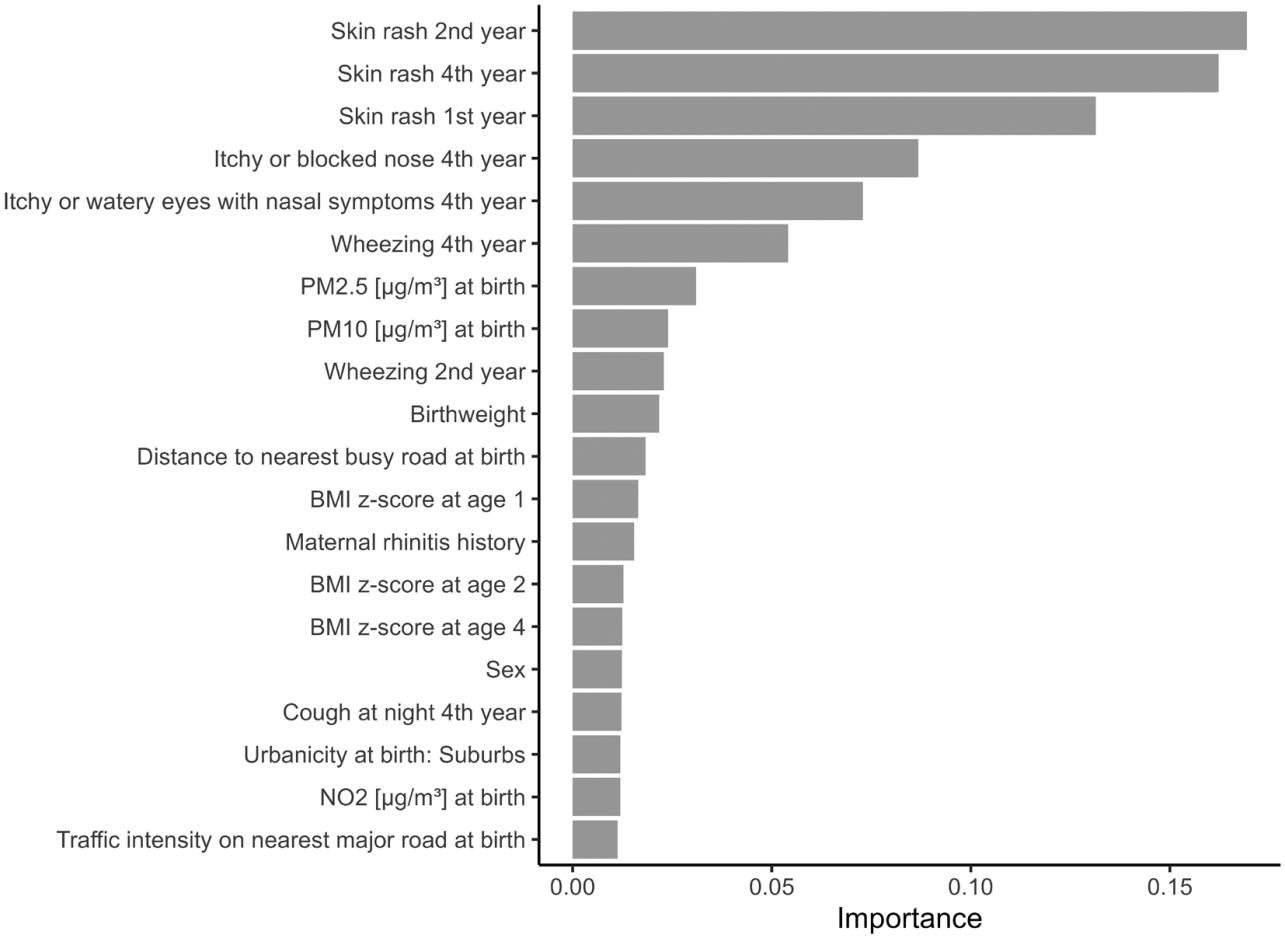
Variable importance for the 20 most important variables, quantifying how useful each predictor is for improving the model's overall prediction accuracy. For variable importance of all predictor variables see Figure S6.

Variable importance ‐ quantifying how useful each predictor is for improving the model's overall prediction accuracy ‐ was calculated as the average gain in accuracy that the model obtains when incorporating a certain variable into a decision tree, that is, higher values indicate that the variable meaningfully improves the model's performance. Based on that, the most important predictors discovered were: early‐life skin rash; respiratory symptoms including an itchy or blocked nose, itchy or watery eyes with nasal symptoms and wheezing; and air pollution at birth including particulate matter (PM2.5 and PM10) (Figure 4). Additionally, other traffic‐ and air pollution related variables, birthweight, BMI, sex, maternal rhinitis history, coughing at night, and urbanicity at birth were among the 20 most important variables. The distributions of predictor variables stratified by trajectories are presented in Table S6. The correlation structure between predictor variables is shown in Figure S5.

The contributions of individual features on the prediction of the single trajectories are presented in SHAP plots in Figure S7. They show the contribution of a feature to a specific prediction but cannot provide effect sizes, nor do they necessarily imply causality. The absence of skin rash in the second and fourth year of life as well as lower air pollution and maternal rhinitis history contributed to the prediction of the rhinitis trajectory. On the other hand, early skin rash, especially in the second year, as well as the absence of itchy or watery eyes with nasal symptoms contributed to the prediction of the early‐resolving dermatitis trajectory. Itchy or watery eyes with nasal symptoms also decreased the probability to be predicted with the mid‐persisting dermatitis trajectory, but together with skin rash and other nasal symptoms they made the prediction of the persisting‐dermatitis + rhinitis trajectory more likely. Additionally, a higher birthweight contributed to the persisting‐dermatitis + rhinitis trajectory as well, whereas lower birthweight, low PM2.5, and low traffic intensity as well as female sex contributed to the prediction of mid‐persisting dermatitis. Wheezing, especially in the fourth year, together with an itchy or blocked nose positively contributed to the multimorbid trajectory, as did high PM2.5 values.

### 3.4 Sub‐analysis including polygenic risk scores as predictors

In the sub‐analysis adding PRS for asthma, allergic rhinitis, atopic dermatitis and any allergy to the predictor set, 1358 participants from GINIplus and 751 from LISA were available (Tables S7 and S8). The multiclass AUC for training data was slightly improved at 0.88 compared to an analysis on the same sample without using PRS variables (AUC = 0.86) (Figures S8 and S9). PRS were among the factors with a comparably high variable importance (Figure S10). However, when evaluating the prediction model in test data, the AUC was not improved at 0.64 with and without PRS (Figures S8 and S9).

## 4 DISCUSSION

In this study, we implemented a machine learning approach to predict allergic disease trajectories from birth until the age of 15 years by using a comprehensive set of early life factors. Our approach yields moderate classification success varying between trajectories with an overall multiclass AUC of 0.80 in training and 0.69 in external test data, and a macro‐averaged sensitivity of 0.26 and specificity of 0.89.

This is, to the best of our knowledge, the first and only study predicting this complex outcome comprising the interplay of different allergic diseases over a long period of time instead of using a single allergic disease at one certain time point as outcome. Shared underlying mechanisms and high comorbidity between the different allergic diseases suggest investigating them jointly to consider time dependence and allergic multimorbidity. However, the similarity and overlap in symptoms, medical history and other factors across trajectories make this a highly challenging prediction problem. The complexity of our outcome might lead to limited accuracy represented by only moderate prediction success. More precisely, the model struggles with differentiating between the non‐allergic control group, mild or transient phenotypes, while the AUC increases to 0.80 and 0.81 when considering the trajectories describing more severe and persistent phenotypes such as the multimorbid and persisting dermatitis + rhinitis trajectory.

The variable importance provides information on which factors are most relevant for future prediction models. This refers to how the variables contribute to the prediction but does not necessarily imply causal associations. As expected, the presence and combination of early allergic symptoms including skin rash, wheezing and itchy or watery eyes or nasal symptoms like an itchy or blocked nose but also maternal rhinitis history were among the most important predictors of allergic disease trajectories – in line with existing prediction scores relying predominantly on parental history and early symptoms or disease.^12^, ^13^, ^14^ Additionally, birthweight and BMI within the first 4 years of life provided information on allergic disease trajectories, which is consistent with previous studies.^15^, ^39^ Air pollution including PM2.5, PM10 and nitrogen dioxide (NO_2_) as well as distance to the nearest busy road and traffic intensity on the nearest major road at birth, reflecting traffic‐related air pollution, have also been identified as important predictors in line with previous studies on the prediction of childhood atopic dermatitis and allergic rhinitis.^18^ Beyond that, factors like sex and urbanicity also slightly improved the model's ability to differentiate between trajectories. However, given the correlation between some of the factors, the predictors identified as most important might also represent other factors to a certain extent.

Although the overall sensitivity is low in our approach at 0.26, it is comparable to the (stringent) API often used in clinical practice for the prediction of asthma at 6 years with a sensitivity of 0.28.^12^ Still, the AUC of the (stringent) API is 0.62,^13^ lower than our model which reaches an AUC of 0.69 in the test data. While the API only considers wheezing, parental asthma history, atopic dermatitis, and allergic rhinitis history of the child as well as allergic rhinitis and eosinophilia, we used a variety of other relevant early life factors like air pollution^5^ or presence of older siblings.^40^ In comparison, the PARS yields a sensitivity of 0.67 and specificity of 0.79 as well as an AUC of 0.79 when replicated in an independent study which is higher than in our approach.^13^ Farhan et al. predicted asthma at age 18 based on factors during the first 4 years and reached an AUC of 0.65, being slightly lower compared to ours.^14^ Although machine learning approaches are more complex, require more input data and are therefore not as easily applicable as some of the prediction scores, they can clearly improve the performance: Owora et al. showed that conditional inference trees have better prognostic performance than prediction scores^16^: They reach an AUC of 0.85 with a sensitivity of 0.47 and specificity of 0.93 – which is comparable to the performance of our model in the training data with a sensitivity of 0.44 and specificity of 0.90. Owora et al. did not perform external validation, which is a major limitation, as this usually leads to a reduction of the performance due to overfitting on the training data. Compared to our AUC of 0.80 and sensitivity of 0.44 in training data, Kothalawala et al. predicted asthma at 10 years of age based on perinatal factors and early life symptoms using support vector machines with an AUC of 0.82 and a sensitivity of 0.72.^15^ However, when validated in external data, the sensitivity decreased to 0.55, which is still higher than our model performance with a sensitivity of 0.26 in external test data, but rather low considering the comparably simple prediction outcome. Another study achieved an AUC of 0.83 for predicting atopic dermatitis up to the age of 14 years and 0.84 for allergic rhinitis with tree‐based models^18^ using personal characteristics, biological markers, and environmental factors as predictors – without performing external validation, limiting external validity.

Associations between PRS for allergic diseases and trajectories have been shown before, independent of adjustment for family history.^8^ Consequently, the performance of our prediction in the training data improved when including PRS in the set of predictors, with the PRS consistently being among the variables with higher importance. Due to limited availability of genetic data, the sample size for the test dataset is relatively small, leading to generally lower AUC values. However, the AUC in the test data did not increase on addition of genetic information, which may indicate overfitting in model training. Beyond that, genetic data may not further improve prediction performance, as was also shown by van Breugel et al.^41^ One might also speculate that the predictive potential of genetics is partially captured by parental allergies in the predictors.

There are several limitations to our approach. Early‐life skin rash symptoms used as a predictor overlap with information on atopic dermatitis diagnosis in the first years of life, which was used for building the trajectories. As many children experienced skin rash symptoms early in life with subsequent remission, this may have improved the performance in the early‐resolving dermatitis trajectory. Further, the low sensitivity limits the ability of the prediction model to correctly recognize individuals belonging to a certain trajectory. It is important to consider that even though the trajectories being predicted have been replicated in an independent study, they are not yet established and widely used as this is an emerging field of research. Therefore, the outcome itself contains some uncertainty which potentially contributes to the overall low sensitivity. Ultimately, it is important to acknowledge that this study cannot yet provide a reliable individual‐level prediction in a clinical setting due to only moderate performance, restricted calibration and limited clinical utility. However, it can provide valuable insights into how the complex interplay between allergic diseases might be considered for future studies and which factors are relevant for the different trajectories. The results of this study can indicate which factors help the model's prediction accuracy but do not provide information about which factors are important in a causal sense. Additionally, predictors identified in this study may not be generalizable to other populations, especially those of non‐European ancestry, or to settings with different environmental exposures such as some low‐ and middle‐income country settings.

An important strength of this study is the use of a multinomial prediction approach considering allergic multimorbidity compared to existing approaches mostly focusing on only one allergic disease, respectively. Further, the two independent longitudinal birth cohort studies allow the use of one study for model training and one for external validation. Performing the validation of the model in an external study which is slightly different compared to the training data (e.g., regarding urbanization), enables the performance in a real‐world scenario to be assessed. This study sets an example for the further development of the prediction of allergic diseases without focusing on one specific disease or one specific time point, by making use of a variety of easily accessible early life factors and machine learning methods. Our approach shows that despite the complexity of the prediction task, the use of machine learning methods enabled moderate success when predicting allergic disease trajectories from birth until adolescence using only early life factors. It could therefore be widely applicable and should be tested in more data. Combining the use of early life and perinatal factors with molecular or clinical data may further improve the prediction success. Furthermore, future work should aim to differentiate between atopic and non‐atopic asthma as they reflect different pathomechanisms and consequently, might differ regarding the role of early‐life predictors.

In conclusion, our study yields a prediction success comparable to established asthma prediction scores like the API, simultaneously accounting for multiple allergic disease trajectories. This could serve as a basis for the development of more advanced approaches to predict allergic disease trajectories. Insights into which factors contribute to the prediction of which disease trajectories could not only indicate different biological pathways between the trajectories but also enhance the development of targeted prevention strategies.

## AUTHOR CONTRIBUTIONS

Miriam Leskien conducted the analyses and was involved in methodology, visualization, writing—original draft preparation, and writing—review and editing. Martin Scheerer contributed to formal analysis, methodology, visualization, and writing—review and editing. Elisabeth Thiering contributed to conceptualization, methodology, supervision, and writing—review and editing. Sara Kress was involved in formal analysis, data curation and writing—review and editing. Claire Coffey provided support in methodological questions and writing—review and editing. Dietrich Berdel, Andrea von Berg, Carl‐Peter Bauer, Monika Gappa, Joachim Heinrich, Sibylle Koletzko, Tamara Schikowski involved in data curation and writing—review and editing. Berthold Koletzko and Annette Peters contributed to conceptualization, methodology, and writing—review and editing. Marie Standl supervised the project performing conceptualization, data curation, funding acquisition, methodology and writing—review and editing. All authors revised and commented on the final manuscript version.

## FUNDING INFORMATION

The GINIplus study was mainly supported for the first 3 years of the Federal Ministry for Education, Science, Research and Technology (interventional arm) and Helmholtz Zentrum Munich (former GSF) (observational arm). The 4, 6, 10, and 15 year follow‐up examinations of the GINIplus study were covered from the respective budgets of the 5 study centres (Helmholtz Zentrum Munich (former GSF), Research Institute at Marien‐Hospital Wesel, LMU Munich, TU Munich and from 6 years onwards also from IUF‐Leibniz Research‐Institute for Environmental Medicine at the University of Düsseldorf) and a grant from the Federal Ministry for Environment (IUF Düsseldorf, FKZ 20462296). Further, the 15 year follow‐up examination of the GINIplus study was supported by the Commission of the European Communities, the 7th Framework Program: MeDALL project, and as well by the companies Mead Johnson and Nestlé. The LISA study was mainly supported by grants from the Federal Ministry for Education, Science, Research and Technology and in addition from Helmholtz Zentrum Munich (former GSF), Helmholtz Centre for Environmental Research‐UFZ, Leipzig, Research Institute at Marien‐Hospital Wesel, Pediatric Practice, Bad Honnef for the first 2 years. The 4, 6, 10, and 15 year follow‐up examinations of the LISA study were covered from the respective budgets of the involved partners (Helmholtz Zentrum Munich (former GSF), Helmholtz Centre for Environmental Research‐UFZ, Leipzig, Research Institute at Marien‐Hospital Wesel, Pediatric Practice, Bad Honnef, IUF–Leibniz‐Research Institute for Environmental Medicine at the University of Düsseldorf) and in addition by a grant from the Federal Ministry for Environment (IUF Düsseldorf, FKZ 20462296). Further, the 15‐year follow‐up examination of the LISA study was supported by the Commission of the European Communities, the 7th Framework Program: MeDALL project. This project has received funding from the European Research Council (ERC) under the European Union's Horizon 2020 research and innovation programme (grant agreement No. 949906). The IUF is funded by the federal and state governments–the Ministry of Culture and Science of North Rhine‐Westphalia (MKW) and the Federal Ministry of Research, Technology and Space (BMFTR).

## CONFLICT OF INTEREST STATEMENT

Monika Gappa receives lecture fees from Nestlé and participates on an advisory board both not related to this work. Sibylle Koletzko reports grants from Mead Johnson Company during the conduct of the study and personal fees from Nestlé Nutrition and Danone outside the submitted work. Berthold Koletzko reports grants from the German Ministry of Education and Research, the German Ministry of Health, the Innovation Committee of the German Joint Federal Committee of Physicians and Public Health Insurances, the European Commission, the Else Kröner Fresenius Foundation as well as Budenheim, Danone, DGC, Hipp, Nestlé and Reckitt outside the submitted work. He has a leadership role in the European Academy of Pediatrics, the Child Health Foundation, the Biomedical Alliance in Europe and the United European Gastroenterology. Claire Coffey reports a personal grant from the Wellcome Trust & Health Data Research UK outside the submitted work. All other authors have no conflicts of interest to declare.

## Supporting information


**Data S1:** Supplemental methods.
**Table S1:** List of candidate predictors with variable description.
**Table S2:** Distribution of predictor variables by cohort (after missing imputation).
**Table S3:** Metrics of prediction performance in the test dataset: Sensitivity, specificity, positive, and negative likelihood ratio.
**Table S4:** Distributions of observed and predicted trajectories and their overlaps.
**Table S5:** Probability thresholds, sensitivity and specificity identified by the Closest Point Method per trajectory.
**Table S6:** Distribution of predictor variables by allergic disease trajectory (after missing imputation).
**Table S7:** Distribution of predictor variables by cohort (after missing imputation) in the subsample for the analysis including PRS.
**Table S8:** Frequencies of allergic disease trajectories in the sub‐sample for the analysis including PRS.
**Figure S1:** Receiver operator characteristic (ROC) curves per trajectory in the complete test dataset.
**Figure S2:** Calibration plot assessing model calibration in test data using flexible Locally Estimated Scatterplot Smoothing (LOESS) curves–before recalibration. Red lines correspond to the ideal curve with intercept = 0 and slope = 1, gray lines correspond to the flexible LOESS calibration curves, and gray shaded areas represent 95% confidence intervals. Calibration intercept and slope are given along with 95% confidence intervals.
**Figure S3:** Calibration plot assessing model calibration in test data using flexible Locally Estimated Scatterplot Smoothing (LOESS) curves–after recalibration. Red lines correspond to the ideal curve with intercept = 0 and slope = 1, gray lines correspond to the flexible LOESS calibration curves, and gray shaded areas represent 95% confidence intervals. Calibration intercept and slope are given along with 95% confidence intervals.
**Figure S4:** Decision curve analysis for assessment of clinical utility. The net benefit was calculated for each trajectory using a one‐vs‐all approach. “Treat none” (gray line at *y* = 0) and “Treat all” (dashed gray lines per trajectory) curves are shown for comparison.
**Figure S5:** Correlation heatmap based on Spearman correlation coefficients between all predictor variables (in training data).
**Figure S6:** Variable importance for all predictor variables, quantifying how useful each predictor is for improving the model’s overall prediction accuracy. It is calculated as the average gain in accuracy that the model obtains when incorporating a certain variable into a decision tree, that is, higher values mean that the variable meaningfully improves the model’s performance.
**Figure S7:** Contribution of the 20 most important features to the prediction of the individual trajectories in the test set represented by SHAP feature values per trajectory (A–G). SHAP values above 0 positively impact the prediction, that is, increase the probability of the prediction of the trajectory, SHAP values below 0 have a negative impact, that is, decrease the probability of the prediction of this trajectory. Larger SHAP values represent larger contributions to the models prediction.
**Figure S8:** Receiver operator characteristic (ROC) curves per trajectory in training and test dataset, with PRS added to the predictor set, shown as mean performance complemented by 95% confidence intervals derived from performances across iterations over cross‐validation folds and replicates for training and bootstraps for test data. Sample size per trajectory is shown for the training dataset followed by the values for the test dataset in brackets. Mean AUC values are presented for training and test data. Multiclass AUC training (test): 0.88 (0.64).
**Figure S9:** Receiver operator characteristic (ROC) curves per trajectory in training and test dataset when PRS were not included, shown as mean performance complemented by 95% confidence intervals derived from performances across iterations over cross‐validation folds and replicates for training and bootstraps for test data. Sample size per trajectory is shown for the training dataset followed by the values for the test dataset in brackets. Mean AUC values are presented for training and test data. Multiclass AUC training (test): 0.86 (0.64).
**Figure S10:** Variable importance for sub‐analysis including polygenic risk scores, for all predictor variables, quantifying how useful each predictor is for improving the model’s overall prediction accuracy.
