# Supplementary material for: Prediction of allergic disease trajectories from birth up to adolescence
Source: Pediatr Allergy Immunol. 2026 Apr 12;37(4):e70341. doi: 10.1111/pai.70341 (PMC13071128; doi:10.1111/pai.70341)
Supplement: Supplementary file 1 — Data S1: Supplemental methods. Table S1: List of candidate predictors with variable description. Table S2: Distribution of predictor variables by cohort (after missing imputation). Table S3: Metrics of prediction performance in the test dataset: Sensitivity, specificity, positive, and negative likelihood ratio. Table S4: Distributions of observed and predicted trajectories and their overlaps. Table S5: Probability thresholds, sensitivity and specificity identified by the Closest Point Method per trajectory. Table S6: Distribution of predictor variables by allergic disease trajectory (after missing imputation). Table S7: Distribution of predictor variables by cohort (after missing imputation) in the subsample for the analysis including PRS. Table S8: Frequencies of allergic disease trajectories in the sub‐sample for the analysis including PRS. Figure S1: Receiver operator characteristic (ROC) curves per trajectory in the complete test dataset. Figure S2: Calibration plot assessing model calibration in test data using flexible Locally Estimated Scatterplot Smoothing (LOESS) curves–before recalibration. Red lines correspond to the ideal curve with intercept = 0 and slope = 1, gray lines correspond to the flexible LOESS calibration curves, and gray shaded areas represent 95% confidence intervals. Calibration intercept and slope are given along with 95% confidence intervals. Figure S3: Calibration plot assessing model calibration in test data using flexible Locally Estimated Scatterplot Smoothing (LOESS) curves–after recalibration. Red lines correspond to the ideal curve with intercept = 0 and slope = 1, gray lines correspond to the flexible LOESS calibration curves, and gray shaded areas represent 95% confidence intervals. Calibration intercept and slope are given along with 95% confidence intervals. Figure S4: Decision curve analysis for assessment of clinical utility. The net benefit was calculated for each trajectory using a one‐vs‐all approach. “Treat none” (gray li [file PAI-37-e70341-s001.docx]

Supplement

Prediction of allergic disease trajectories from birth up to adolescence.

Miriam Leskien, MSc ^1,2,3^,

Martin Scheerer, MSc ^1,2^,

Elisabeth Thiering, PhD ^1,4^,

Sara Kress, PhD ^5^,

Claire Coffey, PhD ^1^,

Dietrich Berdel, MD ^6^,

Andrea von Berg, MD ^6^,

Carl-Peter Bauer, MD ^7^,

Monika Gappa, MD ^8^,

Joachim Heinrich, PhD ^9,10,11^,

Sibylle Koletzko, MD ^4,12^,

Tamara Schikowski, PhD ^5^,

Berthold Koletzko, MD ^4^,

Annette Peters, PhD ^1,13^,

Marie Standl, PhD ^1,3,11^

^1^ Institute of Epidemiology, Helmholtz Zentrum München - German Research Center for Environmental Health, Neuherberg, Germany

^2^ Institute for Medical Information Processing, Biometry, and Epidemiology, LMU Munich, Munich, Germany

^3^ German Center for Child and Adolescent Health (DZKJ), Munich, Germany

^4^ Department of Paediatrics, Dr. von Hauner Children's Hospital, LMU University Hospital, Munich, Germany.

^5^ IUF - Leibniz Research Institute for Environmental Medicine, Düsseldorf, Germany

^6^ Research Institute, Department of Pediatrics, Marien-Hospital Wesel, Wesel, Germany

^7^ Department of Pediatrics, Technical University of Munich, Munich, Germany

^8^ Evangelisches Krankenhaus Düsseldorf, Children's Hospital, Düsseldorf, Germany

^9^ Institute and Clinic for Occupational, Social and Environmental Medicine, University Hospital, LMU Munich, Munich, Germany

^10^ Allergy and Lung Health Unit, Melbourne School of Population and Global Health, The University of Melbourne, Melbourne, Australia

^11^ German Center for Lung Research (DZL)

^12^ Department of Pediatrics, Gastroenterology and Nutrition, School of Medicine Collegium Medicum, University of Warmia and Mazury, Olsztyn, Poland

^13^ Chair of Epidemiology, Ludwig-Maximilians-Universität München, Munich, Germany

**Corresponding author:**

Marie Standl, PhD, Institute of Epidemiology, Helmholtz Zentrum München – German Research Center for Environmental Health, Ingolstädter Landstr. 1, 85764 Neuherberg, Germany. E-mail: [marie.standl@helmholtz-munich.de](mailto:marie.standl@helmholtz-munich.de)

**Supplemental Methods**

**Imputation of missing values:**

All variables included in the final predictor subset had less than 10% missing values in both studies except for daycare in LISA with a missing proportion of 13%. Predictive mean matching was applied for numeric data, logistic regression for binary variables, and multinomial and ordered logit for categorical and ordered categorical variables^1^.

**XGBoost machine learning model:**

The XGBoost machine learning method is part of the gradient boosting framework. Gradient boosting starts with one simple decision tree and sequentially adds a new decision tree that corrects the error made by the previous trees. The algorithm stops when either the predetermined number of trees is reached, or the models’ performance is not improving anymore. A decision tree consists of a root node which includes the total population and is then split into sub-nodes, so-called decision nodes, by a certain feature. Each decision node can then be split again by another feature. The following parameters of the model are determined before fitting the prediction model by performing hyperparameter tuning: the number of splits (tree depth), the number of predictors randomly sampled at each split, the minimum number of data points needed in a node for a split to be made, the number of decision trees and the learning rate which controls how much impact each new tree has on the prediction. Compared to the standard gradient boosting algorithm, XGBoost includes regularization techniques and tree pruning which limits the depth of decision trees to prevent models from overfitting the data and getting too complex. It also supports parallel processing leading to faster computation times. The XGBoost method is described in detail by Chen et al.^2^ and explained for non-experts elsewhere^3^.

**Variable importance:**

XGBoost also provides a built-in feature importance which is quantifying the relative influence of each feature on the predicted outcome. It is defined as the average improvement in accuracy brought by a feature when used in splits.

**Genetic data:**

Genetic data was derived from blood (or saliva) samples collected at 6 and 10 years at the Munich study center and at 6, 10 and 15 years at the Wesel study center. The Affymetrix Chip 5.0 and 6.0 (Thermo Fisher, USA) was used in Munich, and the Infinium Global Screening Array GSA v2 MD (GRCh37/hg19) in Wesel. Individuals with a call rate below 95%, heterozygosity outside mean +/- 4sd, a failure of the sex check or a failure of the similarity quality control using MDS analysis based on IBS, were excluded. Variants with a call rate below 95%, a MAF <0.01 or a HWE p-value <0.00001 were excluded. Genotype imputation was done using the HRC version 1.1^4^ on the Michigan imputation server^5^.

**Polygenic Risk Scores (PRS):**

Genome-wide significant SNPs from previously published genome-wide association studies (GWAS) on asthma^6^, allergic rhinitis^7^, atopic dermatitis^8^, and any allergy^9^ were used to construct PRS. SNP dosages were extracted for Munich and Wesel individuals and were weighted by the respective effect size for each participant. Variants with a low imputation quality (R2 < 0.4) were excluded. Effect alleles were aligned to the ones reported in the GWAS. Polygenic risk scores (PRS) were calculated as the weighted sum of GWAS effect estimates and individual dosage information and transformed into z-scores.

More information on the genetic data and the PRS calculation is provided elsewhere^10,11^.

Table S1: List of candidate predictors with variable description

| **Variable Definition** | **Variable Coding** |
| --- | --- |
| Sex | male, female |
| Study center location | Munich, Wesel |
|  |  |
| **Parental factors:** |  |
| Parental education | low, medium, high |
| Maternal asthma history | no, yes |
| Maternal rhinitis history | no, yes |
| Maternal atopic dermatitis history | no, yes |
| Paternal asthma history | no, yes |
| Paternal rhinitis history | no, yes |
| Paternal atopic dermatitis history | no, yes |
|  |  |
| **Perinatal factors:** |  |
| Mode of birth delivery: caesarean section | no, yes |
| Weight at birth | numeric |
| Exclusive breastfeeding during the first 4 months | no, yes |
| Maternal smoking during pregnancy (2^nd^ and 3^rd^ trimester) | no, yes |
| Mother exposed to smoke during pregnancy | no, yes |
|  |  |
| **Environment or lifestyle factors:** |  |
| Older siblings | no, yes |
| BMI z-score^a^ at age 1 | numeric |
| BMI z-score^a^ at age 2 | numeric |
| BMI z-score^a^ at age 4 | numeric |
| Indoor smoking at home during the first 4 months | no, yes |
| Indoor smoking at home in the 2^nd^ year | no, yes |
| Indoor smoking at home in the 3^rd^ or 4^th^ year | no, yes |
| Gas for cooking used during 1^st^ year | no, yes |
| Mold inside home during 1^st^ year (excluding food) | no, yes |
| NO2 (nitrogen dioxide) [µg/m³] at birth | numeric |
| PM2.5 (particulate matter with diameter 2.5µm or smaller) [µg/m³] at birth | numeric |
| PM10 (particulate matter with diameter 10µm or smaller) [µg/m³] at birth | numeric |
| Distance to the nearest road [m] at birth | numeric |
| Distance to the nearest road [m] with over 5000 vehicles/day at birth | numeric |
| Traffic intensity on nearest road [100 vehicles/day*m] at birth | numeric |
| Traffic intensity on nearest major road [1000 vehicles/day*m] at birth | numeric |
| Urbanicity of the home address at birth | city, towns/suburbs, rural |
| Contact with other children in the 1^st^ year (categorized based on tertiles) | low, medium, high |
| Daycare center visited in the 1^st^ or 2^nd^ year | no, yes |
| Dog at home in the 1^st^ year | no, yes |
| Dog at home in the 2^nd^ year | no, yes |
| Dog at home in the 4^th^ year | no, yes |
| Cat at home in the 1^st^ year | no, yes |
| Cat at home in the 2^nd^ year | no, yes |
| Cat at home in the 4^th^ year | no, yes |
|  |  |
| **Allergy symptoms:** |  |
| Skin rash^b^ in the 1^st^ year | no, yes |
| Skin rash^b^ in the 2^nd^ year | no, yes |
| Skin rash^b^ in the 4^th^ year | no, yes |
| Wheezing in the 1^st^ year | no, yes |
| Wheezing in the 2^nd^ year | no, yes |
| Wheezing in the 4^th^ year | no, yes |
| Itchy or blocked nose without presence of cold in the 1^st^ year | no, yes |
| Itchy or blocked nose without presence of cold in the 2^nd^ year | no, yes |
| Itchy or blocked nose without presence of cold in the 4^th^ year | no, yes |
| Airway infection in the 1^st^ year | no, yes |
| Airway infection in the 2^nd^ year | no, yes |
| Airway infection in the 4^th^ year | no, yes |
| Dry cough at night without cold/bronchitis in the 1^st^ year | no, yes |
| Dry cough at night without cold/bronchitis in the 2^nd^ year | no, yes |
| Dry cough at night without cold/bronchitis in the 4^th^ year | no, yes |
| Itchy or watery eyes together with nasal symptoms in the 1^st^ year | no, yes |
| Itchy or watery eyes together with nasal symptoms in the 4^th^ year | no, yes |
|  |  |

^a^ based on the 2006 WHO child growth standards^12^

^b^ at relevant regions of the body (bends of the elbows, back of the knees, wrists or ankles, face, neck)

Table S2: Distribution of predictor variables by cohort (after missing imputation).

|  | **Training dataset (GINIplus)** | **Test dataset (LISA)** | **Overall** |
| --- | --- | --- | --- |
|  | (N=3277) | (N=1369) | (N=4646) |
| Male sex | 1644 (50.2%) | 720 (52.6%) | 2364 (50.9%) |
| Study center |  |  |  |
| Munich | 1649 (50.3%) | 1147 (83.8%) | 2796 (60.2%) |
| Wesel | 1628 (49.7%) | 222 (16.2%) | 1850 (39.8%) |
|  |  |  |  |
| **Parental factors:** |  |  |  |
| Parental education |  |  |  |
| Low | 269 (8.2%) | 69 (5.0%) | 338 (7.3%) |
| Medium | 974 (29.7%) | 259 (18.9%) | 1233 (26.5%) |
| High | 2034 (62.1%) | 1041 (76.0%) | 3075 (66.2%) |
| Maternal asthma history | 272 (8.3%) | 106 (7.7%) | 378 (8.1%) |
| Maternal rhinitis history | 930 (28.4%) | 464 (33.9%) | 1394 (30.0%) |
| Maternal atopic dermatitis history | 375 (11.4%) | 165 (12.1%) | 540 (11.6%) |
| Paternal asthma history | 236 (7.2%) | 84 (6.1%) | 320 (6.9%) |
| Paternal rhinitis history | 831 (25.4%) | 450 (32.9%) | 1281 (27.6%) |
| Paternal atopic dermatitis history | 186 (5.7%) | 80 (5.8%) | 266 (5.7%) |
|  |  |  |  |
| **Perinatal factors:** |  |  |  |
| Mode of birth delivery: caesarean section | 712 (21.7%) | 258 (18.8%) | 970 (20.9%) |
| Weight at birth: Mean (SD) | 3470 (469) | 3450 (436) | 3460 (460) |
| Exclusive breastfeeding during the first 4 months | 1786 (54.5%) | 833 (60.8%) | 2619 (56.4%) |
| Maternal smoking during pregnancy (2^nd^ and 3^rd^ trimester) | 328 (10.0%) | 149 (10.9%) | 477 (10.3%) |
| Mother exposed to smoke during pregnancy | 696 (21.2%) | 223 (16.3%) | 919 (19.8%) |
|  |  |  |  |
| **Environment or lifestyle factors:** |  |  |  |
| Older siblings | 1558 (47.5%) | 494 (36.1%) | 2052 (44.2%) |
| BMI z-score^a^ at 1 year of age: Mean (SD) | 0.06 (1.01) | -0.08 (0.97) | 0.02 (1.00) |
| BMI z-score^a^ at 2 years of age: Mean (SD) | 0.25 (1.03) | 0.23 (0.97) | 0.24 (1.01) |
| BMI z-score^a^ at 4 years of age: Mean (SD) | 0.09 (0.96) | 0.07 (0.91) | 0.08 (0.95) |
| Indoor smoking at home during the first 4 months | 671 (20.5%) | 210 (15.3%) | 881 (19.0%) |
| Indoor smoking at home in the 2nd year | 854 (26.1%) | 330 (24.1%) | 1184 (25.5%) |
| Indoor smoking at home in the 3rd or 4th year | 1150 (35.1%) | 308 (22.5%) | 1458 (31.4%) |
| Gas for cooking used during first year | 205 (6.3%) | 107 (7.8%) | 312 (6.7%) |
| Mold inside home during first year (excluding food) | 804 (24.5%) | 289 (21.1%) | 1093 (23.5%) |
| NO2 [µg/m³] at birth: Median [Min, Max] | 22.5 [11.5, 62.8] | 21.5 [11.5, 58.9] | 22.3 [11.5, 62.8] |
| PM2.5 [µg/m³] at birth: Median [Min, Max] | 16.0 [10.9, 21.5] | 13.6 [10.6, 19.1] | 14.3 [10.6, 21.5] |
| PM10 [µg/m³] at birth: Median [Min, Max] | 24.0 [14.8, 34.4] | 20.8 [14.8, 31.7] | 22.2 [14.8, 34.4] |
| Distance to the nearest road [m] at birth |  |  |  |
| Median [Min, Max] | 17.7 [0.01, 555] | 19.5 [0.15, 851] | 18.2 [0.01, 851] |
| Distance to the nearest road [m] with over 5000 vehicles/day at birth |  |  |  |
| Median [Min, Max] | 255 [5.3, 7460] | 198 [5.4, 5570] | 237 [5.3, 7460] |
| Traffic intensity on nearest road [100 vehicles/day*m] at birth |  |  |  |
| Median [Min, Max] | 5.0 [5.0, 822] | 5.0 [5.0, 1340] | 5.0 [5.0, 1340] |
| Traffic intensity on nearest major road [1000 vehicles/day*m] at birth |  |  |  |
| Median [Min, Max] | 12.0 [5.0, 136] | 13.5 [5.1, 136] | 12.5 [5.0, 136] |
| Urbanicity of the home address at birth |  |  |  |
| City | 1302 (39.7%) | 649 (47.4%) | 1951 (42.0%) |
| Suburbs | 1494 (45.6%) | 570 (41.6%) | 2064 (44.4%) |
| Rural | 481 (14.7%) | 150 (11.0%) | 631 (13.6%) |
| Contact with other children in the 1^st^ year |  |  |  |
| Low | 1251 (38.2%) | 493 (36.0%) | 1744 (37.5%) |
| Medium | 1183 (36.1%) | 568 (41.5%) | 1751 (37.7%) |
| High | 843 (25.7%) | 308 (22.5%) | 1151 (24.8%) |
| Daycare in first two years | 159 (4.9%) | 154 (11.2%) | 313 (6.7%) |
| Dog at home in the 1^st^ year | 283 (8.6%) | 98 (7.2%) | 381 (8.2%) |
| Dog at home in the 2^nd^ year | 360 (11.0%) | 110 (8.0%) | 470 (10.1%) |
| Dog at home in the 4^th^ year | 424 (12.9%) | 138 (10.1%) | 562 (12.1%) |
| Cat at home in the 1^st^ year | 267 (8.1%) | 132 (9.6%) | 399 (8.6%) |
| Cat at home in the 2^nd^ year | 338 (10.3%) | 138 (10.1%) | 476 (10.2%) |
| Cat at home in the 4^th^ year | 415 (12.7%) | 159 (11.6%) | 574 (12.4%) |
|  |  |  |  |
| **Allergy symptoms:** |  |  |  |
| Skin rash^b^ in the 1^st^ year | 247 (7.5%) | 292 (21.3%) | 539 (11.6%) |
| Skin rash^b^ in the 2^nd^ year | 414 (12.6%) | 215 (15.7%) | 629 (13.5%) |
| Skin rash^b^ in the 4^th^ year | 360 (11.0%) | 371 (27.1%) | 731 (15.7%) |
| Wheezing in the 1^st^ year | 434 (13.2%) | 310 (22.6%) | 744 (16.0%) |
| Wheezing in the 2^nd^ year | 343 (10.5%) | 313 (22.9%) | 656 (14.1%) |
| Wheezing in the 4^th^ year | 317 (9.7%) | 146 (10.7%) | 463 (10.0%) |
| Itchy or blocked nose without presence of cold  in the 1^st^ year | 365 (11.1%) | 190 (13.9%) | 555 (11.9%) |
| Itchy or blocked nose without presence of cold  in the 2^nd^ year | 221 (6.7%) | 218 (15.9%) | 439 (9.4%) |
| Itchy or blocked nose without presence of cold  in the 4^th^ year | 323 (9.9%) | 136 (9.9%) | 459 (9.9%) |
| Airway infection in the 1^st^ year | 2263 (69.1%) | 1006 (73.5%) | 3269 (70.4%) |
| Airway infection in the 2^nd^ year | 2612 (79.7%) | 1152 (84.1%) | 3764 (81.0%) |
| Airway infection in the 4^th^ year | 2662 (81.2%) | 1094 (79.9%) | 3756 (80.8%) |
| Dry cough at night without cold/bronchitis in the 1^st^ year | 245 (7.5%) | 82 (6.0%) | 327 (7.0%) |
| Dry cough at night without cold/bronchitis in the 2^nd^ year | 242 (7.4%) | 132 (9.6%) | 374 (8.1%) |
| Dry cough at night without cold/bronchitis in the 4^th^ year | 248 (7.6%) | 139 (10.2%) | 387 (8.3%) |
| Itchy or watery eyes together with nasal symptoms  in the 1^st^ year | 97 (3.0%) | 49 (3.6%) | 146 (3.1%) |
| Itchy or watery eyes together with nasal symptoms  in the 4^th^ year | 137 (4.2%) | 53 (3.9%) | 190 (4.1%) |

^a^ based on the 2006 WHO child growth standards^12^

^b^ at relevant regions of the body (bends of the elbows, back of the knees, wrists or ankles, face, neck)

Table S3: Metrics of prediction performance in the test dataset: Sensitivity, specificity, positive and negative likelihood ratio.

|  | **Sensitivity** | **Specificity** | **Positive likelihood ratio** | **Negative likelihood ratio** |
| --- | --- | --- | --- | --- |
| No allergy | 0.74 | 0.61 | 1.9 | 0.43 |
| Intermittently allergic | 0.21 | 0.87 | 1.62 | 0.91 |
| Rhinitis | 0.11 | 0.97 | 3.67 | 0.92 |
| Early-resolving dermatitis | 0.37 | 0.91 | 4.11 | 0.69 |
| Mid-persisting dermatitis | 0.06 | 0.96 | 1.5 | 0.98 |
| Multimorbid | 0.13 | 0.98 | 6.5 | 0.89 |
| Persisting dermatitis + rhinitis | 0.22 | 0.96 | 5.5 | 0.81 |

Table S4: Distributions of observed and predicted trajectories and their overlaps

|  | **Observed Trajectory** | | | | | | |  |
| --- | --- | --- | --- | --- | --- | --- | --- | --- |
| **Predicted Trajectory** | No allergy | Intermittently allergic | Rhinitis | Early-resolving dermatitis | Mid-persisting dermatitis | Multi-morbid | Persisting dermatitis + rhinitis | total |
| No allergy | 641 | 111 | 34 | 14 | 19 | 14 | 4 | 837 |
| Intermittently allergic | 108 | 46 | 14 | 13 | 12 | 4 | 2 | 199 |
| Rhinitis | 22 | 8 | 10 | 3 | 1 | 4 | 1 | 49 |
| Early-resolving dermatitis | 52 | 26 | 12 | 28 | 14 | 6 | 10 | 148 |
| Mid-persisting dermatitis | 26 | 7 | 7 | 4 | 3 | 1 | 3 | 51 |
| Multimorbid | 11 | 3 | 2 | 3 | 1 | 6 | 1 | 27 |
| Persisting dermatitis + rhinitis | 9 | 13 | 10 | 10 | 0 | 10 | 6 | 58 |
| total | 641 | 111 | 34 | 14 | 19 | 14 | 4 | 1369 |

Table S5: Probability thresholds, sensitivity and specificity identified by the Closest Point Method per trajectory

| **Trajectory** | **Probability threshold** | **Sensitivity** | **Specificity** | **Distance derived by Closest Point Method** |
| --- | --- | --- | --- | --- |
| Early-resolving dermatitis | 0.06 | 0.63 | 0.81 | 0.41 |
| Intermittently allergic | 0.21 | 0.60 | 0.53 | 0.62 |
| Mid-persisting dermatitis | 0.04 | 0.67 | 0.74 | 0.42 |
| Multimorbid | 0.04 | 0.73 | 0.78 | 0.35 |
| No allergy | 0.34 | 0.67 | 0.71 | 0.44 |
| Persisting dermatitis + rhinitis | 0.03 | 0.74 | 0.74 | 0.37 |
| Rhinitis | 0.09 | 0.64 | 0.53 | 0.59 |

Table S6: Distribution of predictor variables by allergic disease trajectory (after missing imputation)

|  | **No allergy** | **Intermittently allergic** | **Rhinitis** | **Early-resolving dermatitis** | **Mid-persisting dermatitis** | **Multimorbid** | **Persisting dermatitis + rhinitis** | **Overall** |
| --- | --- | --- | --- | --- | --- | --- | --- | --- |
|  | (N=2700) | (N=826) | (N=352) | (N=288) | (N=186) | (N=184) | (N=110) | (N=4646) |
| Male Sex | 1333 (49.4%) | 444 (53.8%) | 193 (54.8%) | 147 (51.0%) | 68 (36.6%) | 113 (61.4%) | 66 (60.0%) | 2364 (50.9%) |
| Study center |  |  |  |  |  |  |  |  |
| Munich | 1592 (59.0%) | 497 (60.2%) | 237 (67.3%) | 183 (63.5%) | 116 (62.4%) | 96 (52.2%) | 75 (68.2%) | 2796 (60.2%) |
| Wesel | 1108 (41.0%) | 329 (39.8%) | 115 (32.7%) | 105 (36.5%) | 70 (37.6%) | 88 (47.8%) | 35 (31.8%) | 1850 (39.8%) |
|  |  |  |  |  |  |  |  |  |
| **Parental factors:** |  |  |  |  |  |  |  |  |
| Parental education |  |  |  |  |  |  |  |  |
| Low | 193 (7.1%) | 58 (7.0%) | 22 (6.3%) | 23 (8.0%) | 16 (8.6%) | 20 (10.9%) | 6 (5.5%) | 338 (7.3%) |
| Medium | 734 (27.2%) | 199 (24.1%) | 90 (25.6%) | 84 (29.2%) | 43 (23.1%) | 50 (27.2%) | 33 (30.0%) | 1233 (26.5%) |
| High | 1773 (65.7%) | 569 (68.9%) | 240 (68.2%) | 181 (62.8%) | 127 (68.3%) | 114 (62.0%) | 71 (64.5%) | 3075 (66.2%) |
| Maternal asthma history | 163 (6.0%) | 54 (6.5%) | 51 (14.5%) | 33 (11.5%) | 19 (10.2%) | 36 (19.6%) | 22 (20.0%) | 378 (8.1%) |
| Maternal rhinitis history | 665 (24.6%) | 273 (33.1%) | 157 (44.6%) | 104 (36.1%) | 64 (34.4%) | 74 (40.2%) | 57 (51.8%) | 1394 (30.0%) |
| Maternal atopic dermatitis history | 240 (8.9%) | 91 (11.0%) | 48 (13.6%) | 69 (24.0%) | 39 (21.0%) | 28 (15.2%) | 25 (22.7%) | 540 (11.6%) |
| Paternal asthma history | 131 (4.9%) | 64 (7.7%) | 37 (10.5%) | 24 (8.3%) | 17 (9.1%) | 34 (18.5%) | 13 (11.8%) | 320 (6.9%) |
| Paternal rhinitis history | 638 (23.6%) | 239 (28.9%) | 140 (39.8%) | 79 (27.4%) | 62 (33.3%) | 70 (38.0%) | 53 (48.2%) | 1281 (27.6%) |
| Paternal atopic dermatitis history | 125 (4.6%) | 50 (6.1%) | 16 (4.5%) | 28 (9.7%) | 20 (10.8%) | 14 (7.6%) | 13 (11.8%) | 266 (5.7%) |
|  |  |  |  |  |  |  |  |  |
| **Perinatal factors:** |  |  |  |  |  |  |  |  |
| Mode of birth delivery: caesarean section | 542 (20.1%) | 187 (22.6%) | 78 (22.2%) | 58 (20.1%) | 37 (19.9%) | 43 (23.4%) | 25 (22.7%) | 970 (20.9%) |
| Weight at birth: Mean (SD) | 3450 (459) | 3460 (462) | 3470 (450) | 3510 (432) | 3440 (478) | 3490 (473) | 3580 (481) | 3460 (460) |
| Exclusive breastfeeding during the first 4 months | 1527 (56.6%) | 463 (56.1%) | 190 (54.0%) | 176 (61.1%) | 99 (53.2%) | 99 (53.8%) | 65 (59.1%) | 2619 (56.4%) |
| Maternal smoking during pregnancy (2^nd^ and 3^rd^ trimester) | 292 (10.8%) | 77 (9.3%) | 34 (9.7%) | 26 (9.0%) | 23 (12.4%) | 16 (8.7%) | 9 (8.2%) | 477 (10.3%) |
| Mother exposed to smoke during pregnancy | 543 (20.1%) | 153 (18.5%) | 69 (19.6%) | 45 (15.6%) | 47 (25.3%) | 40 (21.7%) | 22 (20.0%) | 919 (19.8%) |
|  |  |  |  |  |  |  |  |  |
| **Environment or lifestyle factors:** |  |  |  |  |  |  |  |  |
| Older siblings | 1254 (46.4%) | 342 (41.4%) | 129 (36.6%) | 132 (45.8%) | 77 (41.4%) | 74 (40.2%) | 44 (40.0%) | 2052 (44.2%) |
| BMI z-score^a^ at 1 year of age:  Mean (SD) | 0.02 (1.00) | -0.07 (0.99) | -0.04 (0.95) | 0.10 (1.05) | 0.15 (0.97) | 0.11 (1.02) | 0.03 (0.97) | 0.02 (1.00) |
| BMI z-score^a^ at 2 years of age:  Mean (SD) | 0.22 (1.02) | 0.25 (1.00) | 0.21 (1.00) | 0.34 (1.01) | 0.37 (1.00) | 0.28 (1.05) | 0.29 (0.98) | 0.24 (1.01) |
| BMI z-score^a^ at 4 years of age:  Mean (SD) | 0.07 (0.96) | 0.06 (0.90) | 0.06 (0.92) | 0.10 (0.96) | 0.14 (0.90) | 0.24 (0.98) | 0.14 (0.86) | 0.08 (0.95) |
| Indoor smoking at home during the first 4 months | 519 (19.2%) | 161 (19.5%) | 58 (16.5%) | 48 (16.7%) | 37 (19.9%) | 34 (18.5%) | 24 (21.8%) | 881 (19.0%) |
| Indoor smoking at home in the 2^nd^ year | 709 (26.3%) | 200 (24.2%) | 85 (24.1%) | 64 (22.2%) | 53 (28.5%) | 46 (25.0%) | 27 (24.5%) | 1184 (25.5%) |
| Indoor smoking at home  in the 3^rd^ or 4^th^ year | 866 (32.1%) | 244 (29.5%) | 108 (30.7%) | 78 (27.1%) | 70 (37.6%) | 57 (31.0%) | 35 (31.8%) | 1458 (31.4%) |
| Gas for cooking used during 1^st^ year | 180 (6.7%) | 49 (5.9%) | 23 (6.5%) | 25 (8.7%) | 11 (5.9%) | 13 (7.1%) | 11 (10.0%) | 312 (6.7%) |
| Mold inside home during 1^st^ year (excluding food) | 613 (22.7%) | 204 (24.7%) | 84 (23.9%) | 69 (24.0%) | 40 (21.5%) | 44 (23.9%) | 39 (35.5%) | 1093 (23.5%) |
| NO2 [µg/m³] at birth:  Median [Min, Max] | 22.3 [11.5, 61.1] | 22.6 [11.5, 58.9] | 21.8 [11.6, 62.8] | 22.0 [11.5, 54.0] | 22.0 [11.5, 59.8] | 22.4 [11.6, 51.8] | 21.8 [11.5, 37.2] | 22.3 [11.5, 62.8] |
| PM2.5 [µg/m³] at birth:  Median [Min, Max] | 14.3 [10.6, 21.5] | 14.4 [10.9, 21.1] | 13.8 [10.8, 20.7] | 14.1 [11.1, 18.9] | 14.0 [11.4, 21.4] | 15.0 [11.5, 20.7] | 13.9 [11.1, 18.8] | 14.3 [10.6, 21.5] |
| PM10 [µg/m³] at birth:  Median [Min, Max] | 22.3 [14.8, 34.4] | 22.3 [14.8, 34.1] | 21.4 [14.8, 30.4] | 21.9 [14.8, 28.9] | 22.2 [14.8, 31.4] | 23.7 [14.8, 30.9] | 21.2 [14.9, 27.7] | 22.2 [14.8, 34.4] |
| Distance to the nearest road [m] at birth |  |  |  |  |  |  |  |  |
| Mean (SD) | 24.2 (29.0) | 23.9 (19.1) | 23.7 (17.2) | 25.3 (29.2) | 23.6 (20.4) | 23.6 (23.8) | 25.1 (18.7) | 24.2 (26.0) |
| Median [Min, Max] | 18.1 [0.01, 851] | 18.5 [0.57, 206] | 19.2 [0.03, 130] | 18.3 [1.07, 376] | 18.2 [0.01, 192] | 17.4 [0.30, 238] | 20.1 [2.50, 128] | 18.2 [0.01, 851] |
| Distance to the nearest road [m] with over 5000 vehicles/day at birth |  |  |  |  |  |  |  |  |
| Mean (SD) | 469 (729) | 415 (630) | 446 (699) | 416 (569) | 474 (677) | 471 (656) | 439 (717) | 454 (696) |
| Median [Min, Max] | 243 [5.4, 7460] | 219 [7.6, 5990] | 256 [15.9, 6090] | 205 [12.6, 3050] | 247 [7.8, 4670] | 240 [5.3, 4040] | 237 [13.1, 4940] | 237 [5.3, 7460] |
| Traffic intensity on nearest road [100 vehicles/day*m] at birth |  |  |  |  |  |  |  |  |
| Mean (SD) | 19.4 (58.7) | 25.7 (90.2) | 13.0 (36.2) | 18.9 (55.4) | 22.1 (58.5) | 19.2 (42.7) | 16.4 (40.2) | 20.0 (63.2) |
| Median [Min, Max] | 5.0 [5.0, 1340] | 5.0 [5.0, 1340] | 5.0 [5.0, 380] | 5.0 [5.0, 650] | 5.0 [5.0, 390] | 5.0 [5.0, 236] | 5.0 [5.0, 280] | 5.0 [5.0, 1340] |
| Traffic intensity on nearest major road [1000 vehicles/day*m] at birth |  |  |  |  |  |  |  |  |
| Mean (SD) | 16.6 (15.5) | 16.3 (14.4) | 16.8 (16.2) | 16.7 (16.3) | 16.8 (16.2) | 16.6 (18.1) | 18.5 (18.1) | 16.6 (15.6) |
| Median [Min, Max] | 12.2 [5.1, 136] | 12.8 [5.0, 136] | 12.8 [5.0, 136] | 12.1 [5.08, 136] | 13.3 [5.1, 136] | 12.0 [5.1, 131] | 13.2 [5.1, 131] | 12.5 [5.0, 136] |
| Urbanicity of the home address at birth |  |  |  |  |  |  |  |  |
| City | 1130 (41.9%) | 342 (41.4%) | 153 (43.5%) | 123 (42.7%) | 84 (45.2%) | 67 (36.4%) | 52 (47.3%) | 1951 (42.0%) |
| Suburbs | 1179 (43.7%) | 387 (46.9%) | 151 (42.9%) | 133 (46.2%) | 82 (44.1%) | 86 (46.7%) | 46 (41.8%) | 2064 (44.4%) |
| Rural | 391 (14.5%) | 97 (11.7%) | 48 (13.6%) | 32 (11.1%) | 20 (10.8%) | 31 (16.8%) | 12 (10.9%) | 631 (13.6%) |
| Contact to other children in the 1^st^ year (categorized based on tertiles) |  |  |  |  |  |  |  |  |
| Low | 1028 (38.1%) | 288 (34.9%) | 143 (40.6%) | 104 (36.1%) | 66 (35.5%) | 65 (35.3%) | 50 (45.5%) | 1744 (37.5%) |
| Medium | 1001 (37.1%) | 326 (39.5%) | 140 (39.8%) | 110 (38.2%) | 66 (35.5%) | 73 (39.7%) | 35 (31.8%) | 1751 (37.7%) |
| High | 671 (24.9%) | 212 (25.7%) | 69 (19.6%) | 74 (25.7%) | 54 (29.0%) | 46 (25.0%) | 25 (22.7%) | 1151 (24.8%) |
| Daycare in first two years | 169 (6.3%) | 62 (7.5%) | 17 (4.8%) | 28 (9.7%) | 18 (9.7%) | 14 (7.6%) | 5 (4.5%) | 313 (6.7%) |
| Dog at home in the 1^st^ year | 246 (9.1%) | 62 (7.5%) | 15 (4.3%) | 16 (5.6%) | 16 (8.6%) | 18 (9.8%) | 8 (7.3%) | 381 (8.2%) |
| Dog at home in the 2^nd^ year | 304 (11.3%) | 75 (9.1%) | 18 (5.1%) | 22 (7.6%) | 21 (11.3%) | 20 (10.9%) | 10 (9.1%) | 470 (10.1%) |
| Dog at home in the 4^th^ year | 367 (13.6%) | 90 (10.9%) | 27 (7.7%) | 23 (8.0%) | 22 (11.8%) | 23 (12.5%) | 10 (9.1%) | 562 (12.1%) |
| Cat at home in the 1^st^ year | 241 (8.9%) | 75 (9.1%) | 26 (7.4%) | 20 (6.9%) | 17 (9.1%) | 11 (6.0%) | 9 (8.2%) | 399 (8.6%) |
| Cat at home in the 2^nd^ year | 285 (10.6%) | 96 (11.6%) | 27 (7.7%) | 26 (9.0%) | 19 (10.2%) | 13 (7.1%) | 10 (9.1%) | 476 (10.2%) |
| Cat at home in the 4^th^ year | 343 (12.7%) | 118 (14.3%) | 30 (8.5%) | 32 (11.1%) | 19 (10.2%) | 17 (9.2%) | 15 (13.6%) | 574 (12.4%) |
|  |  |  |  |  |  |  |  |  |
| **Allergy symptoms:** |  |  |  |  |  |  |  |  |
| Skin rash^b^ in the 1^st^ year | 135 (5.0%) | 113 (13.7%) | 49 (13.9%) | 120 (41.7%) | 22 (11.8%) | 43 (23.4%) | 57 (51.8%) | 539 (11.6%) |
| Skin rash^b^ in the 2^nd^ year | 145 (5.4%) | 131 (15.9%) | 44 (12.5%) | 163 (56.6%) | 44 (23.7%) | 47 (25.5%) | 55 (50.0%) | 629 (13.5%) |
| Skin rash^b^ in the 4^th^ year | 205 (7.6%) | 128 (15.5%) | 43 (12.2%) | 159 (55.2%) | 75 (40.3%) | 58 (31.5%) | 63 (57.3%) | 731 (15.7%) |
| Wheezing in the 1^st^ year | 373 (13.8%) | 146 (17.7%) | 58 (16.5%) | 55 (19.1%) | 34 (18.3%) | 51 (27.7%) | 27 (24.5%) | 744 (16.0%) |
| Wheezing in the 2^nd^ year | 321 (11.9%) | 131 (15.9%) | 45 (12.8%) | 46 (16.0%) | 30 (16.1%) | 60 (32.6%) | 23 (20.9%) | 656 (14.1%) |
| Wheezing in the 4^th^ year | 181 (6.7%) | 98 (11.9%) | 40 (11.4%) | 31 (10.8%) | 17 (9.1%) | 76 (41.3%) | 20 (18.2%) | 463 (10.0%) |
| Itchy or blocked nose without presence of cold in the 1^st^ year | 274 (10.1%) | 104 (12.6%) | 41 (11.6%) | 46 (16.0%) | 32 (17.2%) | 36 (19.6%) | 22 (20.0%) | 555 (11.9%) |
| Itchy or blocked nose without presence of cold in the 2^nd^ year | 187 (6.9%) | 94 (11.4%) | 40 (11.4%) | 37 (12.8%) | 24 (12.9%) | 33 (17.9%) | 24 (21.8%) | 439 (9.4%) |
| Itchy or blocked nose without presence of cold in the 4^th^ year | 144 (5.3%) | 84 (10.2%) | 66 (18.8%) | 38 (13.2%) | 17 (9.1%) | 63 (34.2%) | 47 (42.7%) | 459 (9.9%) |
| Airway infection in the 1^st^ year | 1854 (68.7%) | 594 (71.9%) | 253 (71.9%) | 207 (71.9%) | 140 (75.3%) | 139 (75.5%) | 82 (74.5%) | 3269 (70.4%) |
| Airway infection in the 2^nd^ year | 2132 (79.0%) | 694 (84.0%) | 300 (85.2%) | 239 (83.0%) | 152 (81.7%) | 151 (82.1%) | 96 (87.3%) | 3764 (81.0%) |
| Airway infection in the 4^th^ year | 2148 (79.6%) | 652 (78.9%) | 306 (86.9%) | 244 (84.7%) | 150 (80.6%) | 159 (86.4%) | 97 (88.2%) | 3756 (80.8%) |
| Dry cough at night without cold/bronchitis in the 1^st^ year | 173 (6.4%) | 51 (6.2%) | 26 (7.4%) | 24 (8.3%) | 14 (7.5%) | 27 (14.7%) | 12 (10.9%) | 327 (7.0%) |
| Dry cough at night without cold/bronchitis in the 2^nd^ year | 187 (6.9%) | 70 (8.5%) | 30 (8.5%) | 16 (5.6%) | 18 (9.7%) | 33 (17.9%) | 20 (18.2%) | 374 (8.1%) |
| Dry cough at night without cold/bronchitis in the 4^th^ year | 152 (5.6%) | 70 (8.5%) | 42 (11.9%) | 33 (11.5%) | 20 (10.8%) | 53 (28.8%) | 17 (15.5%) | 387 (8.3%) |
| Itchy or watery eyes together with nasal symptoms in the 1^st^ year | 61 (2.3%) | 28 (3.4%) | 11 (3.1%) | 11 (3.8%) | 10 (5.4%) | 15 (8.2%) | 10 (9.1%) | 146 (3.1%) |
| Itchy or watery eyes together with nasal symptoms in the 4^th^ year | 36 (1.3%) | 33 (4.0%) | 45 (12.8%) | 10 (3.5%) | 1 (0.5%) | 34 (18.5%) | 31 (28.2%) | 190 (4.1%) |
|  |  |  |  |  |  |  |  |  |

^a^ based on the 2006 WHO child growth standards^12^

^b^ at relevant regions of the body (bends of the elbows, back of the knees, wrists or ankles, face, neck)

Table S7: Distribution of predictor variables by cohort (after missing imputation) in the subsample for the analysis including PRS

|  | **Training dataset (GINIplus)** | **Test dataset (LISA)** | **Overall** |
| --- | --- | --- | --- |
|  | (N=1358) | (N=751) | (N=2109) |
| Male sex | 684 (50.4%) | 340 (45.3%) | 1024 (48.6%) |
| Study center |  |  |  |
| Munich | 750 (55.2%) | 653 (87.0%) | 1403 (66.5%) |
| Wesel | 608 (44.8%) | 98 (13.0%) | 706 (33.5%) |
|  |  |  |  |
| **Parental factors:** |  |  |  |
| Parental education |  |  |  |
| Low | 94 (6.9%) | 35 (4.7%) | 129 (6.1%) |
| Medium | 381 (28.1%) | 130 (17.3%) | 511 (24.2%) |
| High | 883 (65.0%) | 586 (78.0%) | 1469 (69.7%) |
| Maternal asthma history | 128 (9.4%) | 65 (8.7%) | 193 (9.2%) |
| Maternal rhinitis history | 417 (30.7%) | 263 (35.0%) | 680 (32.2%) |
| Maternal atopic dermatitis history | 179 (13.2%) | 93 (12.4%) | 272 (12.9%) |
| Paternal asthma history | 107 (7.9%) | 49 (6.5%) | 156 (7.4%) |
| Paternal rhinitis history | 381 (28.1%) | 264 (35.2%) | 645 (30.6%) |
| Paternal atopic dermatitis history | 91 (6.7%) | 40 (5.3%) | 131 (6.2%) |
|  |  |  |  |
| **Perinatal factors:** |  |  |  |
| Mode of birth delivery: caesarean section | 282 (20.8%) | 142 (18.9%) | 424 (20.1%) |
| Weight at birth: Mean (SD) | 3470 (473) | 3460 (432) | 3460 (459) |
| Exclusive breastfeeding during the first 4 months | 775 (57.1%) | 477 (63.5%) | 1252 (59.4%) |
| Maternal smoking during pregnancy (2^nd^ and 3^rd^ trimester) | 121 (8.9%) | 71 (9.5%) | 192 (9.1%) |
| Mother exposed to smoke during pregnancy | 256 (18.9%) | 116 (15.4%) | 372 (17.6%) |
|  |  |  |  |
| **Environment or lifestyle factors:** |  |  |  |
| Older siblings | 657 (48.4%) | 275 (36.6%) | 932 (44.2%) |
| BMI z-score^a^ at 1 year of age: Mean (SD) | 0.05 (1.00) | -0.10 (0.97) | 0.00 (0.99) |
| BMI z-score^a^ at 2 years of age: Mean (SD) | 0.27 (1.01) | 0.24 (0.95) | 0.26 (0.99) |
| BMI z-score^a^ at 4 years of age: Mean (SD) | 0.07 (0.94) | 0.09 (0.89) | 0.08 (0.92) |
| Indoor smoking at home during the first 4 months | 264 (19.4%) | 107 (14.2%) | 371 (17.6%) |
| Indoor smoking at home in the 2nd year | 339 (25.0%) | 163 (21.7%) | 502 (23.8%) |
| Indoor smoking at home in the 3rd or 4th year | 460 (33.9%) | 154 (20.5%) | 614 (29.1%) |
| Gas for cooking used during first year | 80 (5.9%) | 62 (8.3%) | 142 (6.7%) |
| Mold inside home during first year (excluding food) | 345 (25.4%) | 159 (21.2%) | 504 (23.9%) |
| NO2 [µg/m³] at birth: Median [Min, Max] | 22.2 [11.5, 62.8] | 21.5 [11.5, 58.9] | 22.1 [11.5, 62.8] |
| PM2.5 [µg/m³] at birth: Median [Min, Max] | 14.7 [10.9, 21.5] | 13.5 [10.7, 19.0] | 14.0 [10.7, 21.5] |
| PM10 [µg/m³] at birth: Median [Min, Max] | 22.9 [14.8, 34.4] | 20.7 [14.8, 29.5] | 21.7 [14.8, 34.4] |
| Distance to the nearest road [m] at birth |  |  |  |
| Median [Min, Max] | 17.7 [0.01, 501] | 19.6 [0.14, 376] | 18.4 [0.01, 501] |
| Distance to the nearest road [m] with over 5000 vehicles/day at birth |  |  |  |
| Median [Min, Max] | 247 [5.3, 7150] | 186 [11.9, 5570] | 224 [5.3, 7150] |
| Traffic intensity on nearest road [100 vehicles/day*m] at birth |  |  |  |
| Median [Min, Max] | 5.0 [5.0, 822] | 5.0 [5.0, 1340] | 5.0 [5.0, 1340] |
| Traffic intensity on nearest major road [1000 vehicles/day*m] at birth |  |  |  |
| Median [Min, Max] | 12.1 [5.0, 136] | 13.9 [5.1, 134] | 13.0 [5.0, 136] |
| Urbanicity of the home address at birth |  |  |  |
| City | 538 (39.6%) | 380 (50.6%) | 918 (43.5%) |
| Suburbs | 605 (44.6%) | 297 (39.5%) | 902 (42.8%) |
| Rural | 215 (15.8%) | 74 (9.9%) | 289 (13.7%) |
| Contact with other children in the 1^st^ year |  |  |  |
| Low | 533 (39.2%) | 260 (34.6%) | 793 (37.6%) |
| Medium | 483 (35.6%) | 314 (41.8%) | 797 (37.8%) |
| High | 342 (25.2%) | 177 (23.6%) | 519 (24.6%) |
| Daycare in first two years | 73 (5.4%) | 86 (11.5%) | 159 (7.5%) |
| Dog at home in the 1^st^ year | 110 (8.1%) | 49 (6.5%) | 159 (7.5%) |
| Dog at home in the 2^nd^ year | 141 (10.4%) | 56 (7.5%) | 197 (9.3%) |
| Dog at home in the 4^th^ year | 167 (12.3%) | 63 (8.4%) | 230 (10.9%) |
| Cat at home in the 1^st^ year | 117 (8.6%) | 72 (9.6%) | 189 (9.0%) |
| Cat at home in the 2^nd^ year | 140 (10.3%) | 75 (10.0%) | 215 (10.2%) |
| Cat at home in the 4^th^ year | 169 (12.4%) | 89 (11.9%) | 258 (12.2%) |
|  |  |  |  |
| **Allergy symptoms:** |  |  |  |
| Skin rash^b^ in the 1^st^ year | 119 (8.8%) | 163 (21.7%) | 282 (13.4%) |
| Skin rash^b^ in the 2^nd^ year | 189 (13.9%) | 120 (16.0%) | 309 (14.7%) |
| Skin rash^b^ in the 4^th^ year | 161 (11.9%) | 219 (29.2%) | 380 (18.0%) |
| Wheezing in the 1^st^ year | 183 (13.5%) | 175 (23.3%) | 358 (17.0%) |
| Wheezing in the 2^nd^ year | 150 (11.0%) | 171 (22.8%) | 321 (15.2%) |
| Wheezing in the 4^th^ year | 136 (10.0%) | 81 (10.8%) | 217 (10.3%) |
| Itchy or blocked nose without presence of cold  in the 1^st^ year | 173 (12.7%) | 105 (14.0%) | 278 (13.2%) |
| Itchy or blocked nose without presence of cold  in the 2^nd^ year | 106 (7.8%) | 118 (15.7%) | 224 (10.6%) |
| Itchy or blocked nose without presence of cold  in the 4^th^ year | 149 (11.0%) | 79 (10.5%) | 228 (10.8%) |
| Airway infection in the 1^st^ year | 941 (69.3%) | 576 (76.7%) | 1517 (71.9%) |
| Airway infection in the 2^nd^ year | 1107 (81.5%) | 639 (85.1%) | 1746 (82.8%) |
| Airway infection in the 4^th^ year | 1133 (83.4%) | 602 (80.2%) | 1735 (82.3%) |
| Dry cough at night without cold/bronchitis in the 1^st^ year | 97 (7.1%) | 43 (5.7%) | 140 (6.6%) |
| Dry cough at night without cold/bronchitis in the 2^nd^ year | 93 (6.8%) | 75 (10.0%) | 168 (8.0%) |
| Dry cough at night without cold/bronchitis in the 4^th^ year | 112 (8.2%) | 86 (11.5%) | 198 (9.4%) |
| Itchy or watery eyes together with nasal symptoms  in the 1^st^ year | 44 (3.2%) | 31 (4.1%) | 75 (3.6%) |
| Itchy or watery eyes together with nasal symptoms  in the 4^th^ year | 59 (4.3%) | 29 (3.9%) | 88 (4.2%) |
|  |  |  |  |
| **Polygenic risk scores** |  |  |  |
| PRS Asthma: Mean (SD) | -0.01 (0.98) | 0.03 (1.03) | 0.01 (1.00) |
| PRS Rhinitis: Mean (SD) | -0.03 (0.99) | 0.03 (1.02) | -0.00 (1.00) |
| PRS Eczema: Mean (SD) | -0.00 (0.98) | 0.00 (1.02) | 0.00 (0.99) |
| PRS Any Allergy: Mean (SD) | -0.03 (1.01) | 0.04 (0.98) | -0.00 (1.00) |

^a^ based on the 2006 WHO child growth standards^12^

^b^ at relevant regions of the body (bends of the elbows, back of the knees, wrists or ankles, face, neck)

Table S8: Frequencies of allergic disease trajectories in the sub-sample for the analysis including PRS

|  | **Training dataset (GINIplus)** | **Test dataset (LISA)** | **Overall** |
| --- | --- | --- | --- |
|  | (N=1358) | (N=751) | (N=2109) |
| No allergy | 694 (51.1%) | 458 (61.0%) | 1152 (54.6%) |
| Intermittently allergic | 253 (18.6%) | 125 (16.6%) | 378 (17.9%) |
| Rhinitis | 129 (9.5%) | 54 (7.2%) | 183 (8.7%) |
| Early-resolving dermatitis | 97 (7.1%) | 41 (5.5%) | 138 (6.5%) |
| Mid-persisting dermatitis | 68 (5.0%) | 28 (3.7%) | 96 (4.6%) |
| Multimorbid | 74 (5.4%) | 27 (3.6%) | 101 (4.8%) |
| Persisting dermatitis + rhinitis | 43 (3.2%) | 18 (2.4%) | 61 (2.9%) |

Figure S1: Receiver Operator Characteristic (ROC) curves per trajectory in the complete test dataset.


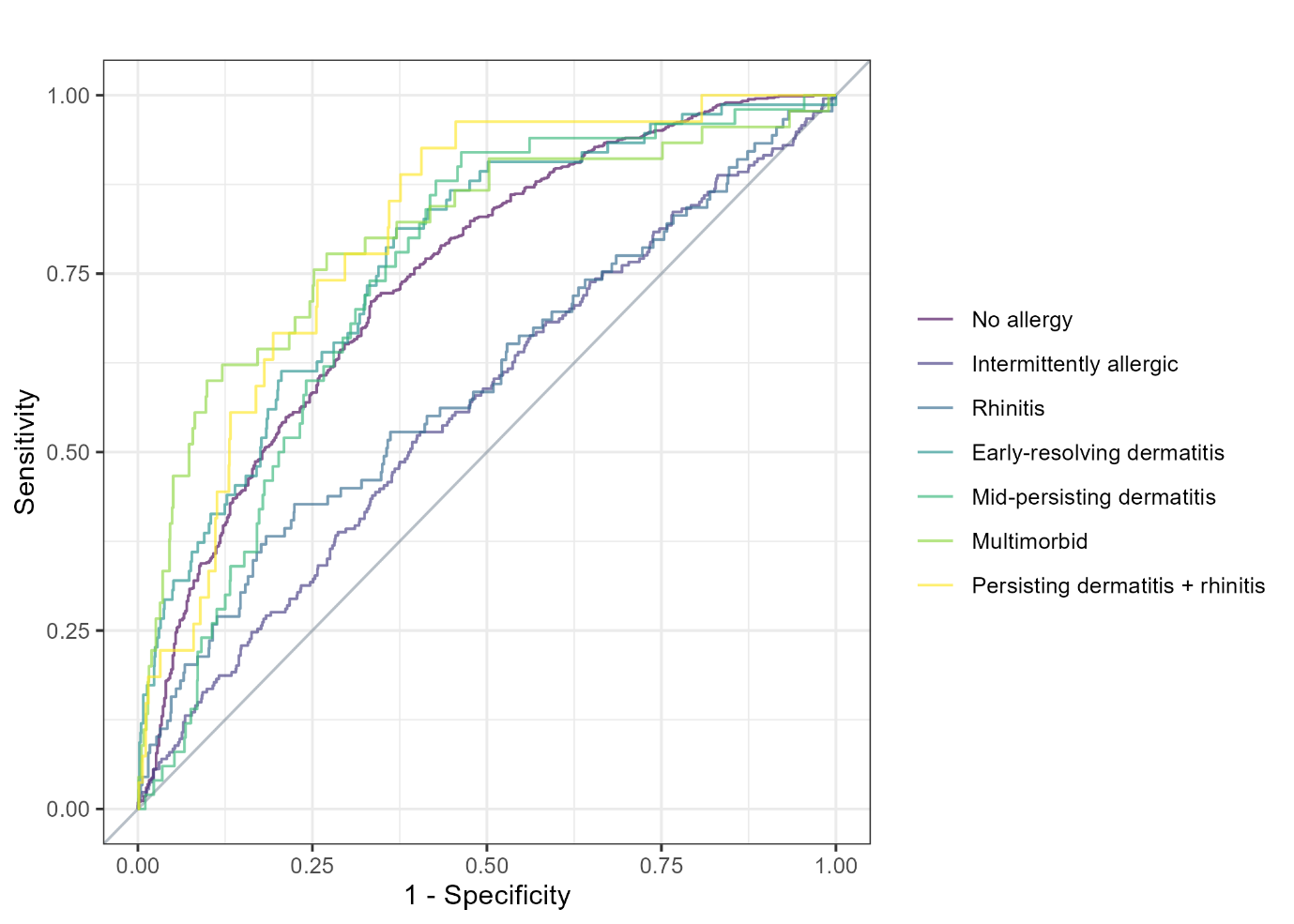


Figure S2: Calibration plot assessing model calibration in test data using flexible Locally Estimated Scatterplot Smoothing (LOESS) curves – before recalibration. Red lines correspond to the ideal curve with intercept = 0 and slope = 1, grey lines correspond to the flexible LOESS calibration curves, and grey shaded areas represent 95% confidence intervals. Calibration intercept and slope are given along with 95% confidence intervals.


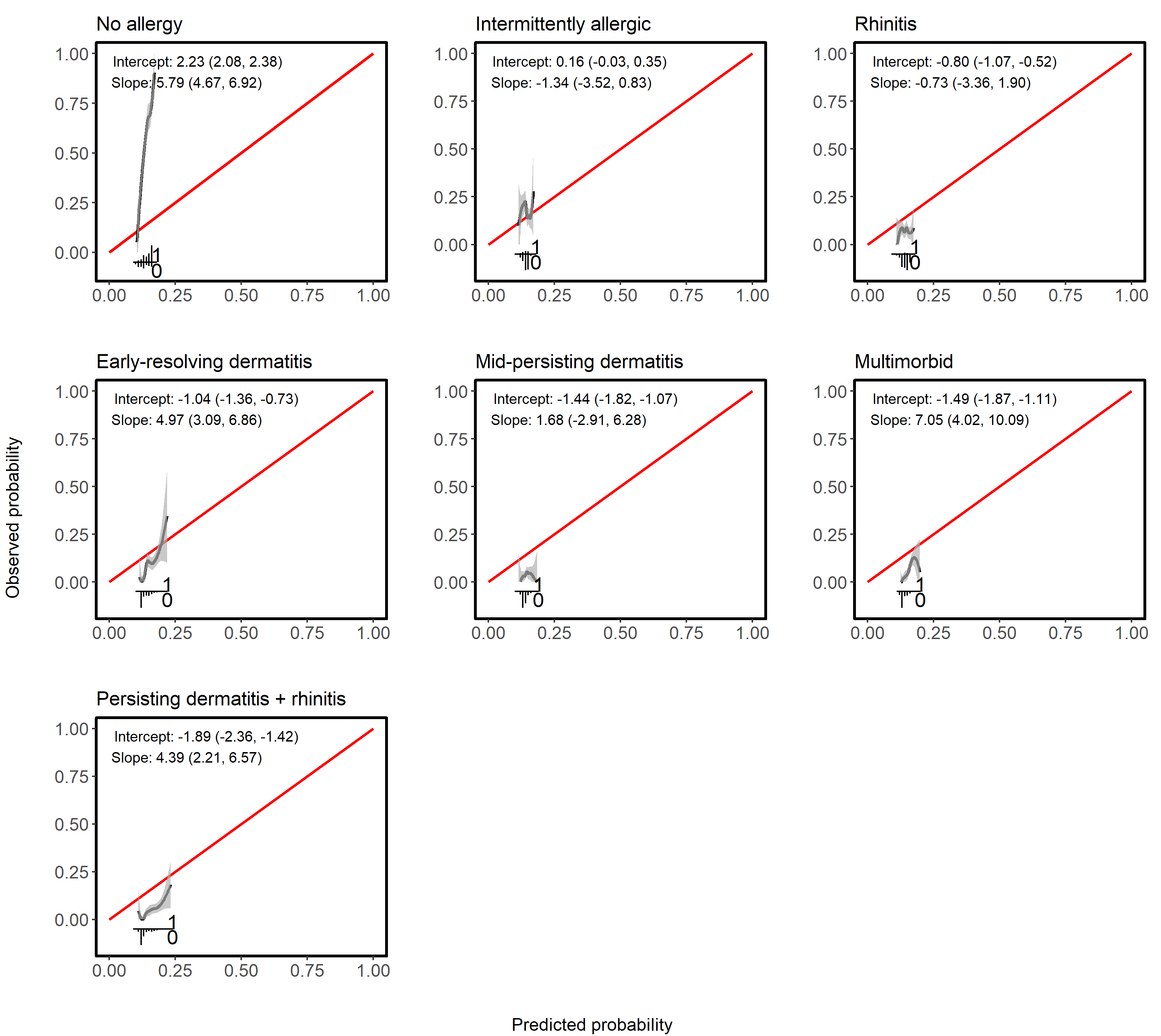


Figure S3: Calibration plot assessing model calibration in test data using flexible Locally Estimated Scatterplot Smoothing (LOESS) curves – after recalibration. Red lines correspond to the ideal curve with intercept = 0 and slope = 1, grey lines correspond to the flexible LOESS calibration curves, and grey shaded areas represent 95% confidence intervals. Calibration intercept and slope are given along with 95% confidence intervals.


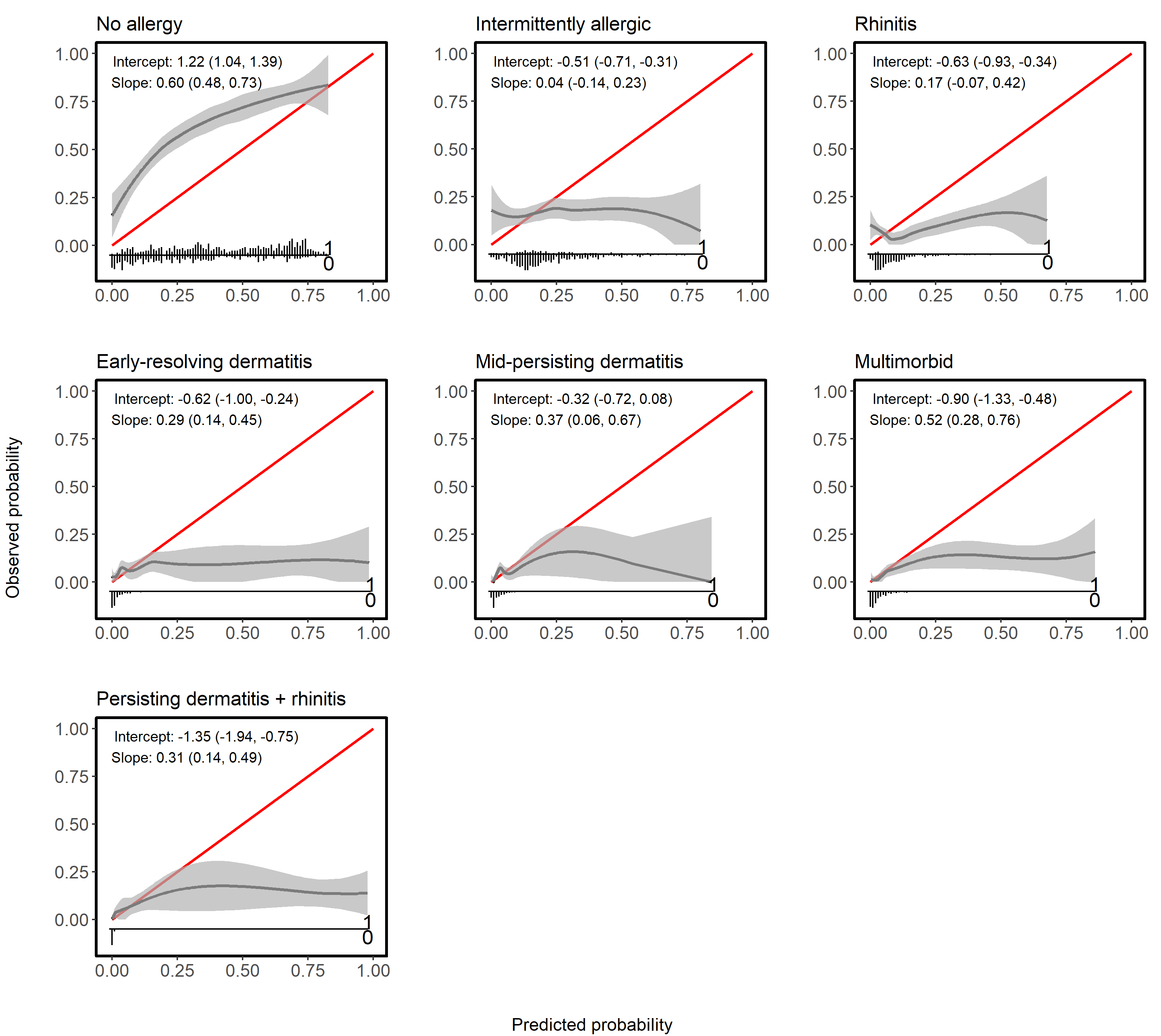


Figure S4: Decision curve analysis for assessment of clinical utility. The net benefit was calculated for each trajectory using a one-vs-all approach. ‘Treat none’ (grey line at y=0) and ‘Treat all’ (dashed grey lines per trajectory) curves are shown for comparison.


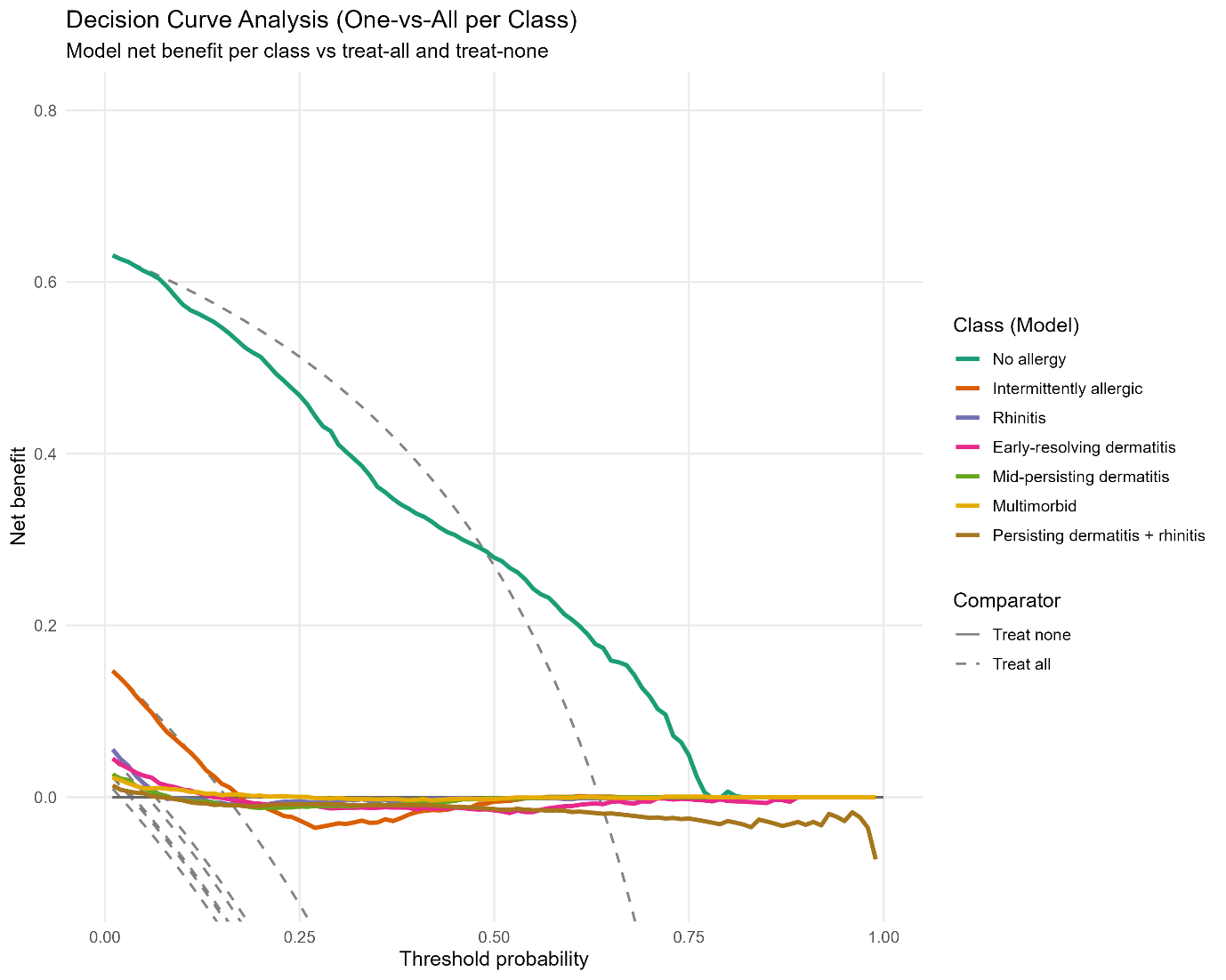


Figure S5: Correlation heatmap based on Spearman correlation coefficients between all predictor variables (in training data)


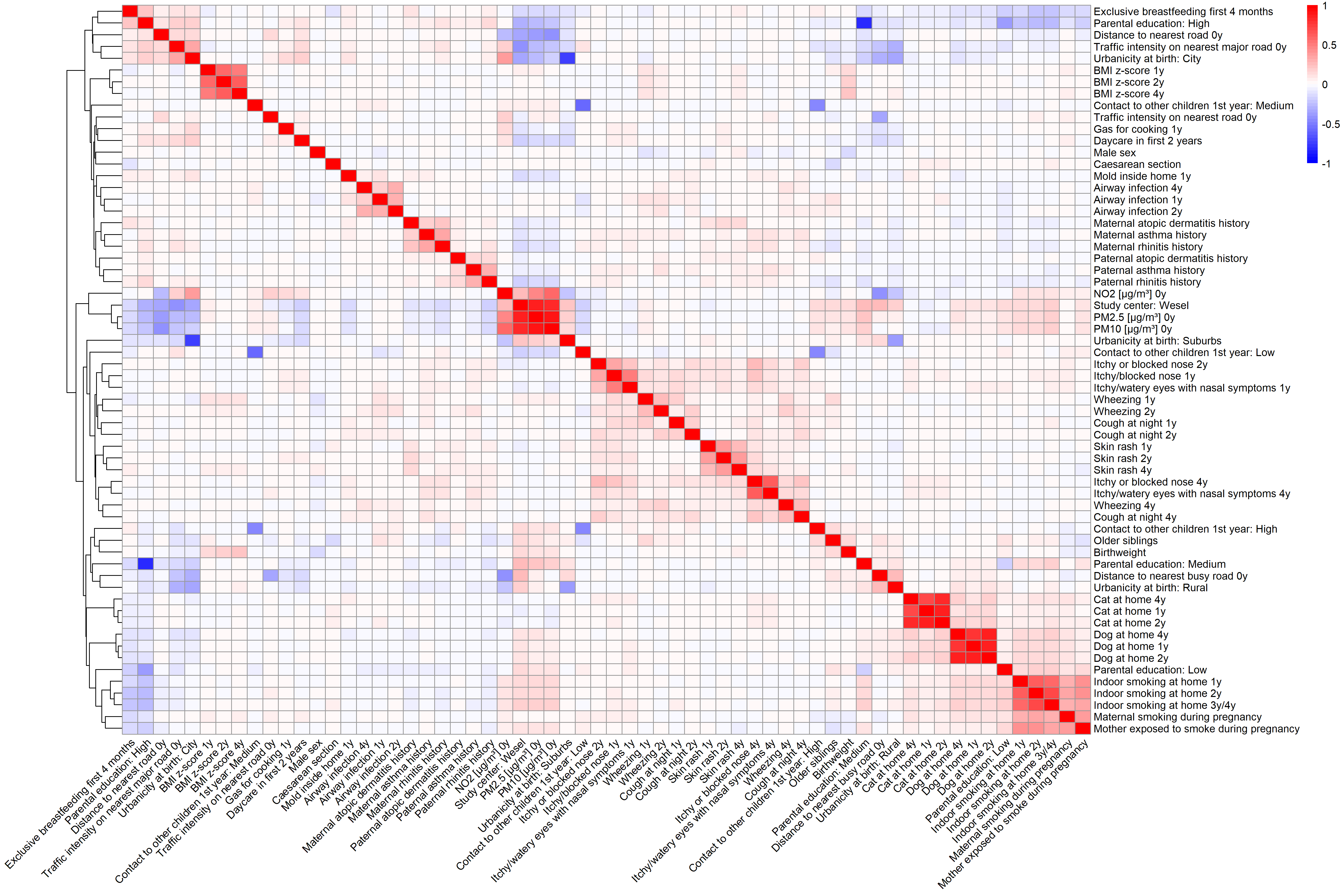


Figure S6: Variable importance for all predictor variables, quantifying how useful each predictor is for improving the model’s overall prediction accuracy. It is calculated as the average gain in accuracy that the model obtains when incorporating a certain variable into a decision tree, i. e. higher values mean that the variable meaningfully improves the model’s performance.


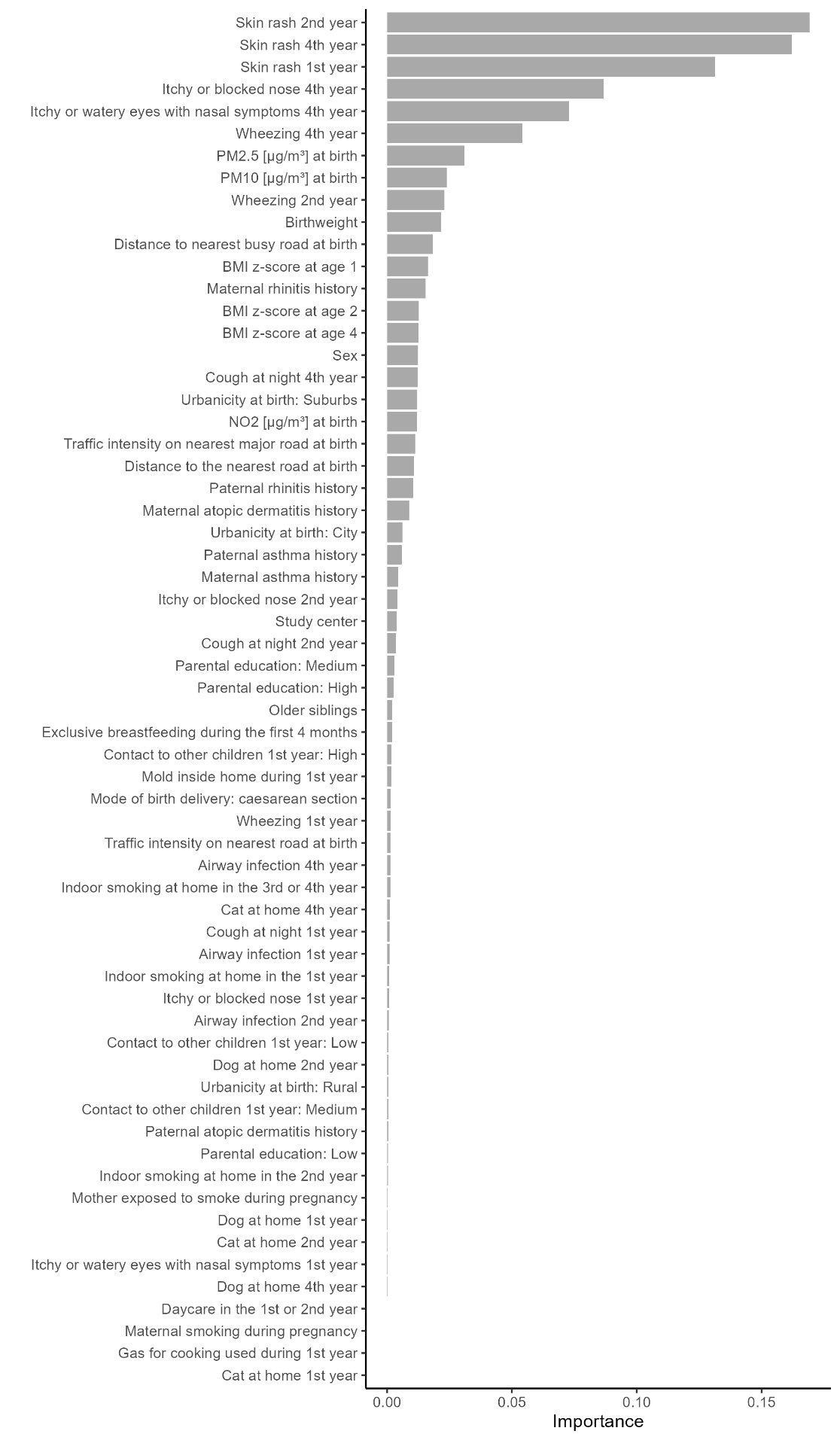


Figure S7: Contribution of the 20 most important variables to the prediction of the individual trajectories in the test set represented by SHAP feature values per trajectory (**A-G**). SHAP values above 0 positively impact the prediction, i. e. increase the probability of the prediction of the trajectory, SHAP values below 0 have a negative impact, i. e. decrease the probability of the prediction of this trajectory. Larger SHAP values represent larger contributions to the models’ prediction.


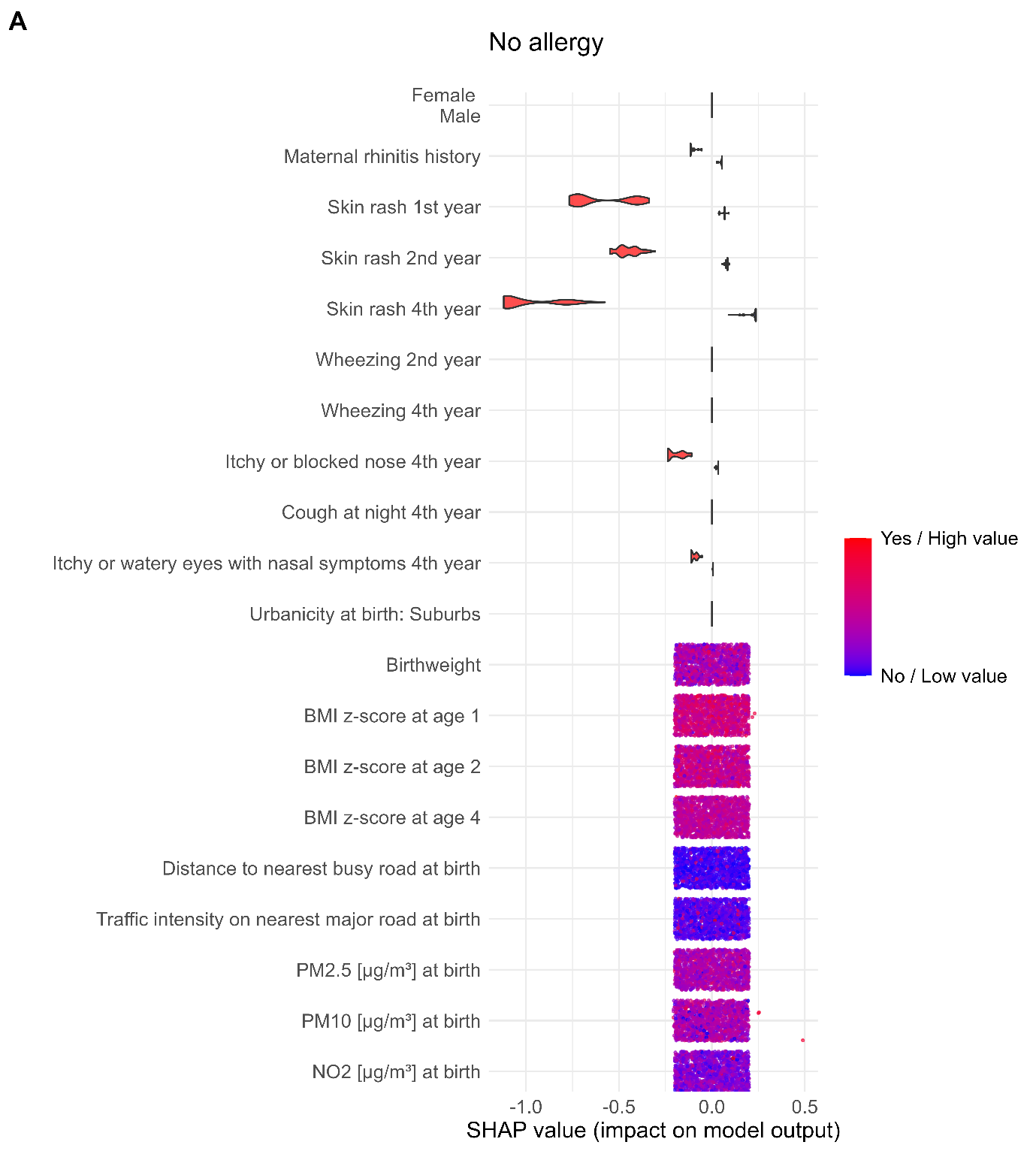


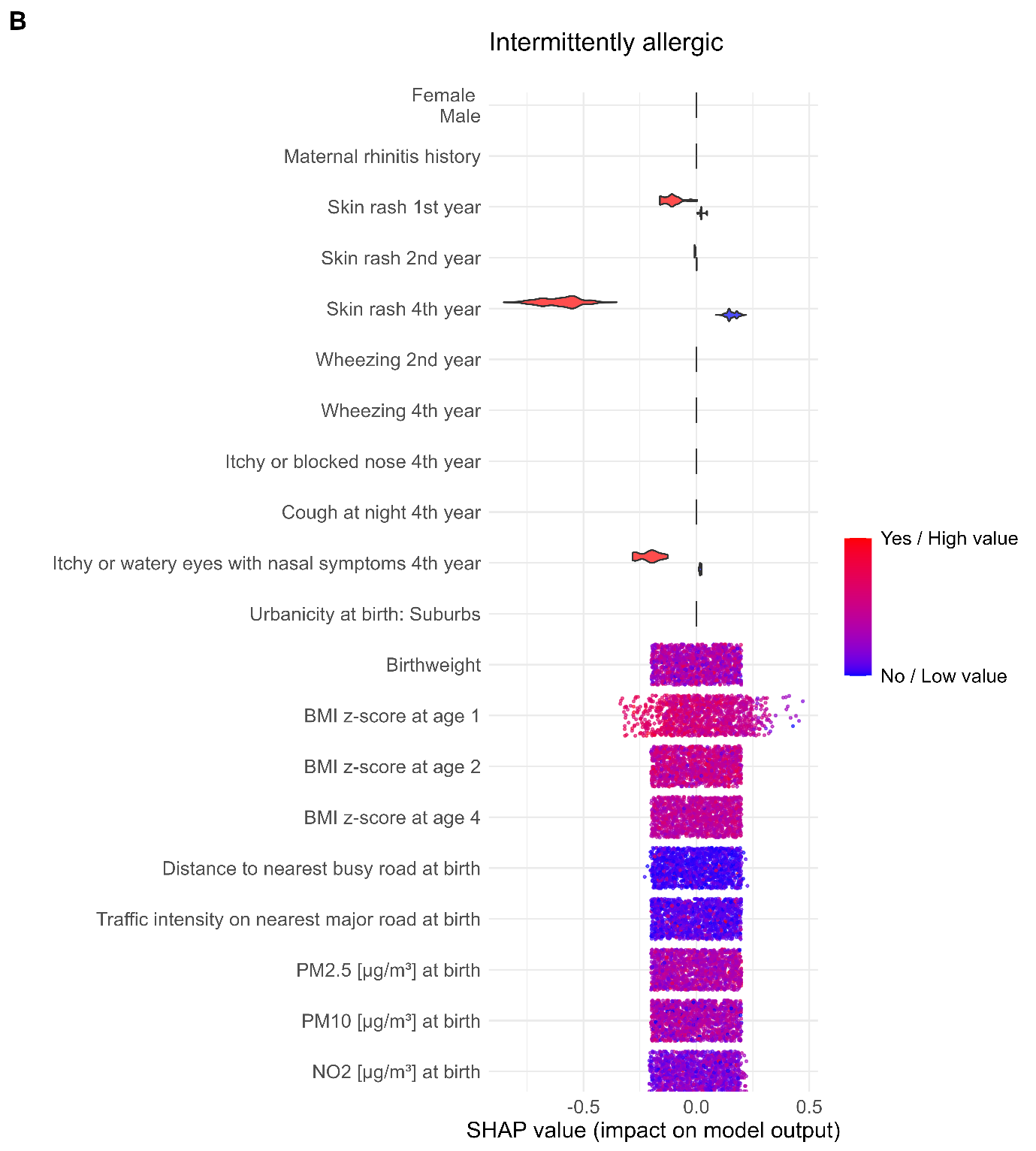


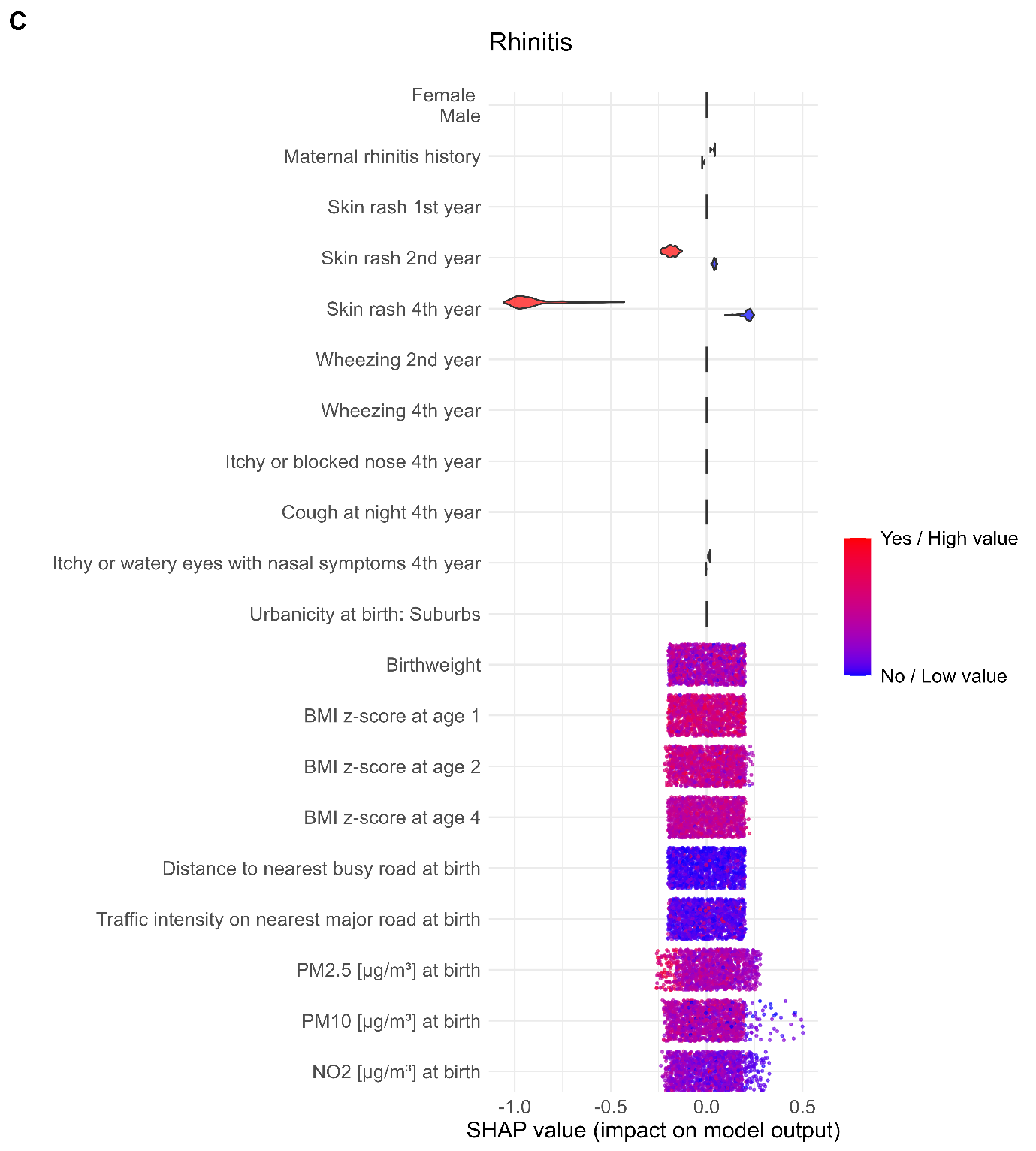


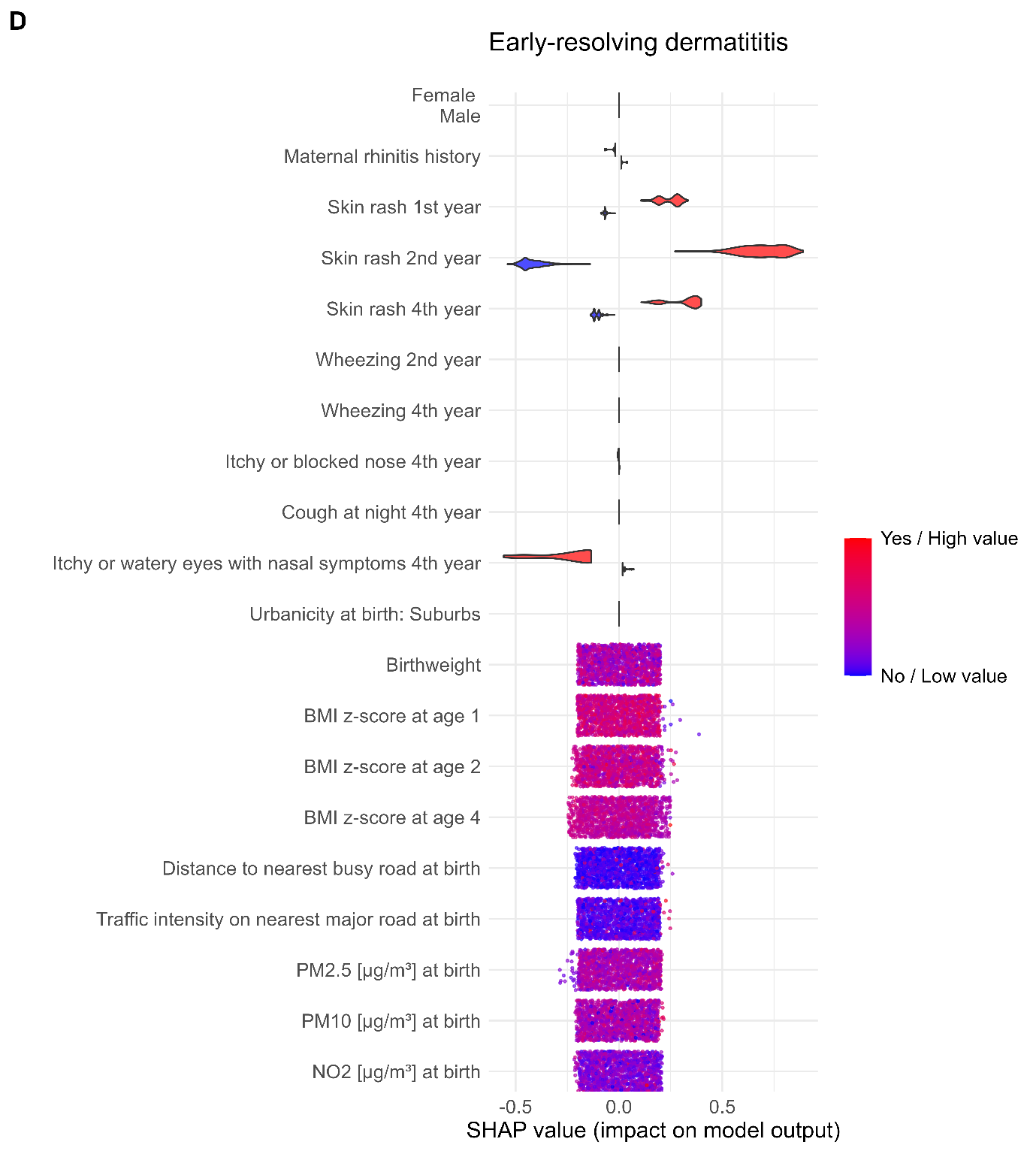


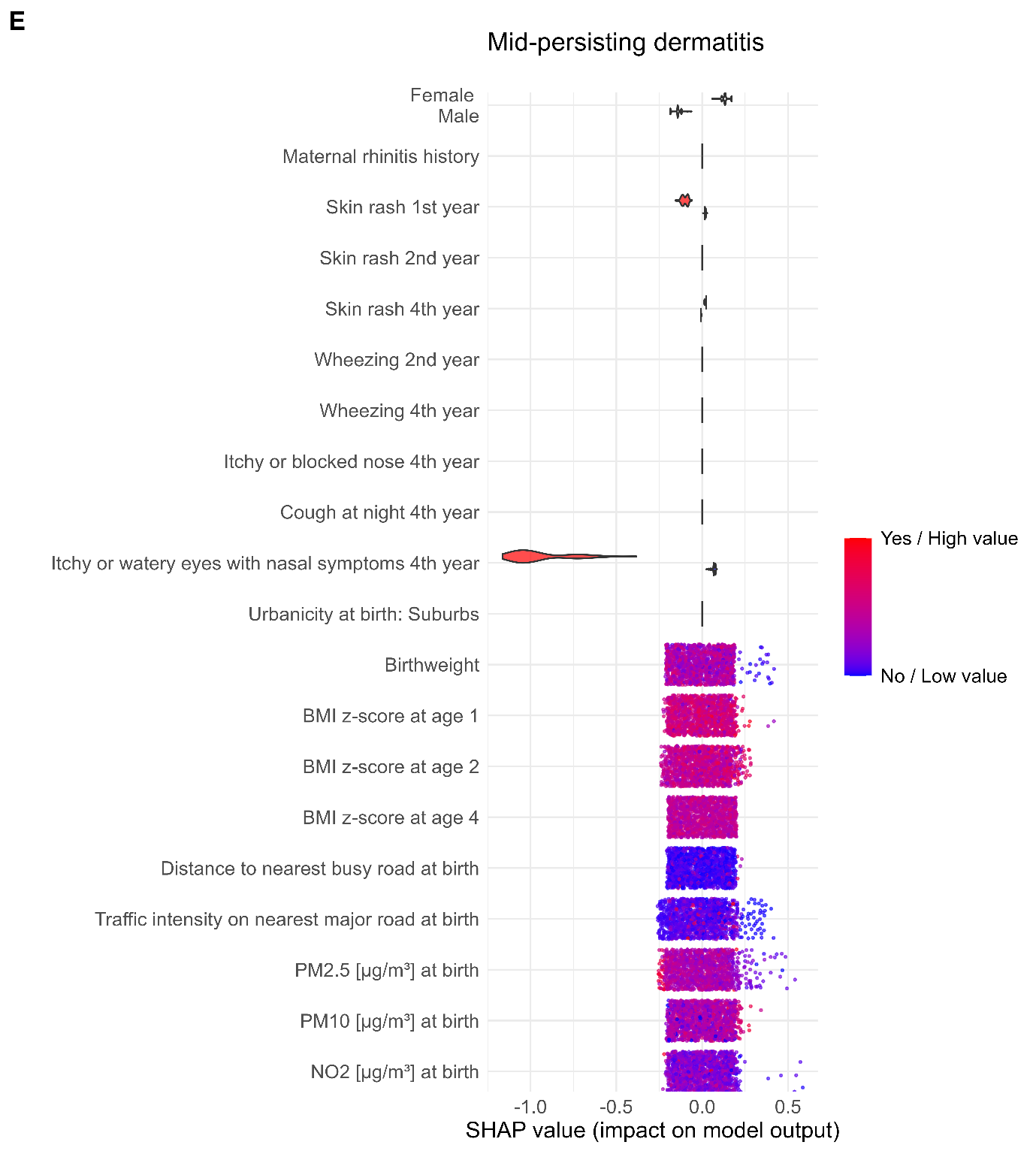


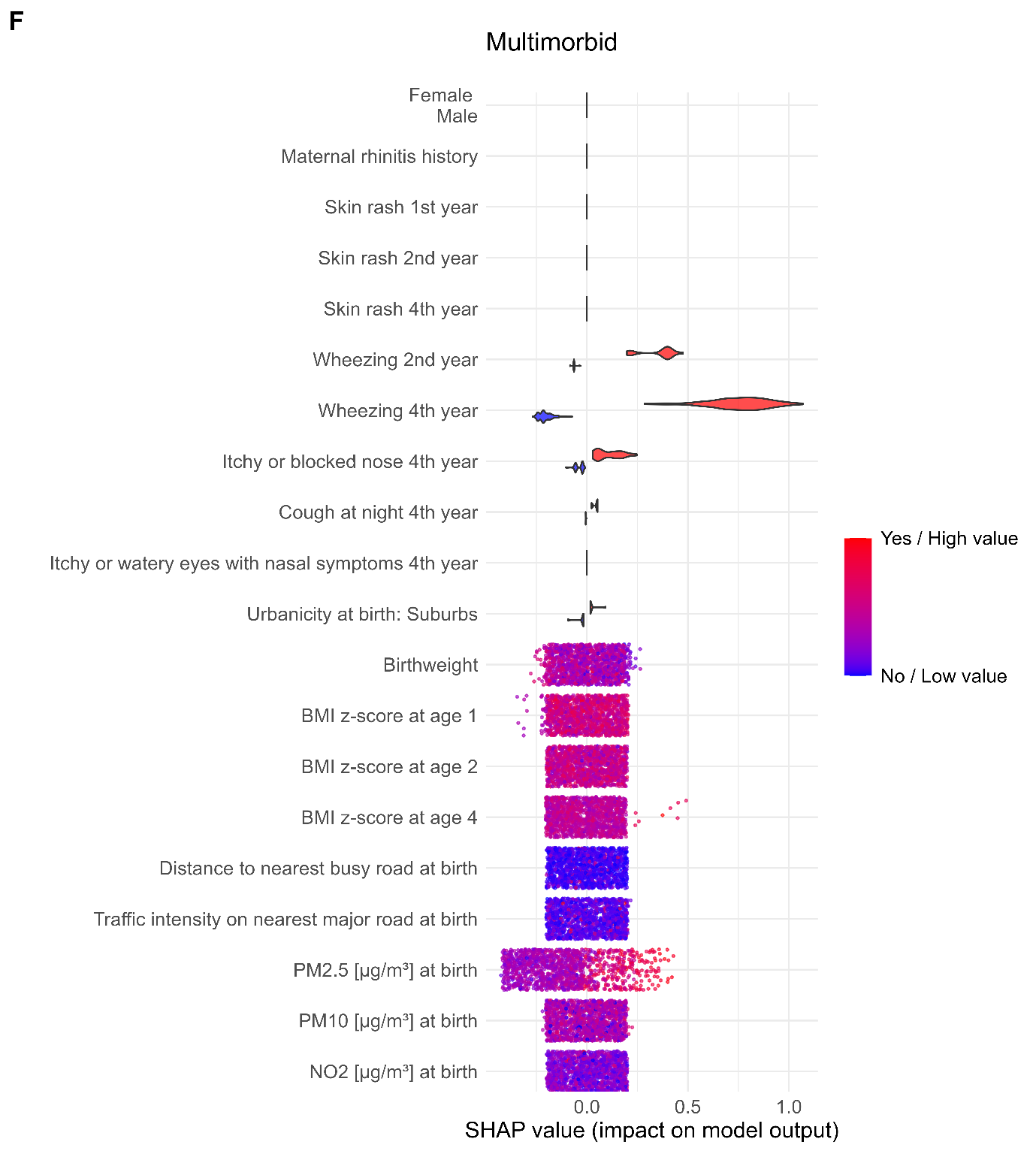


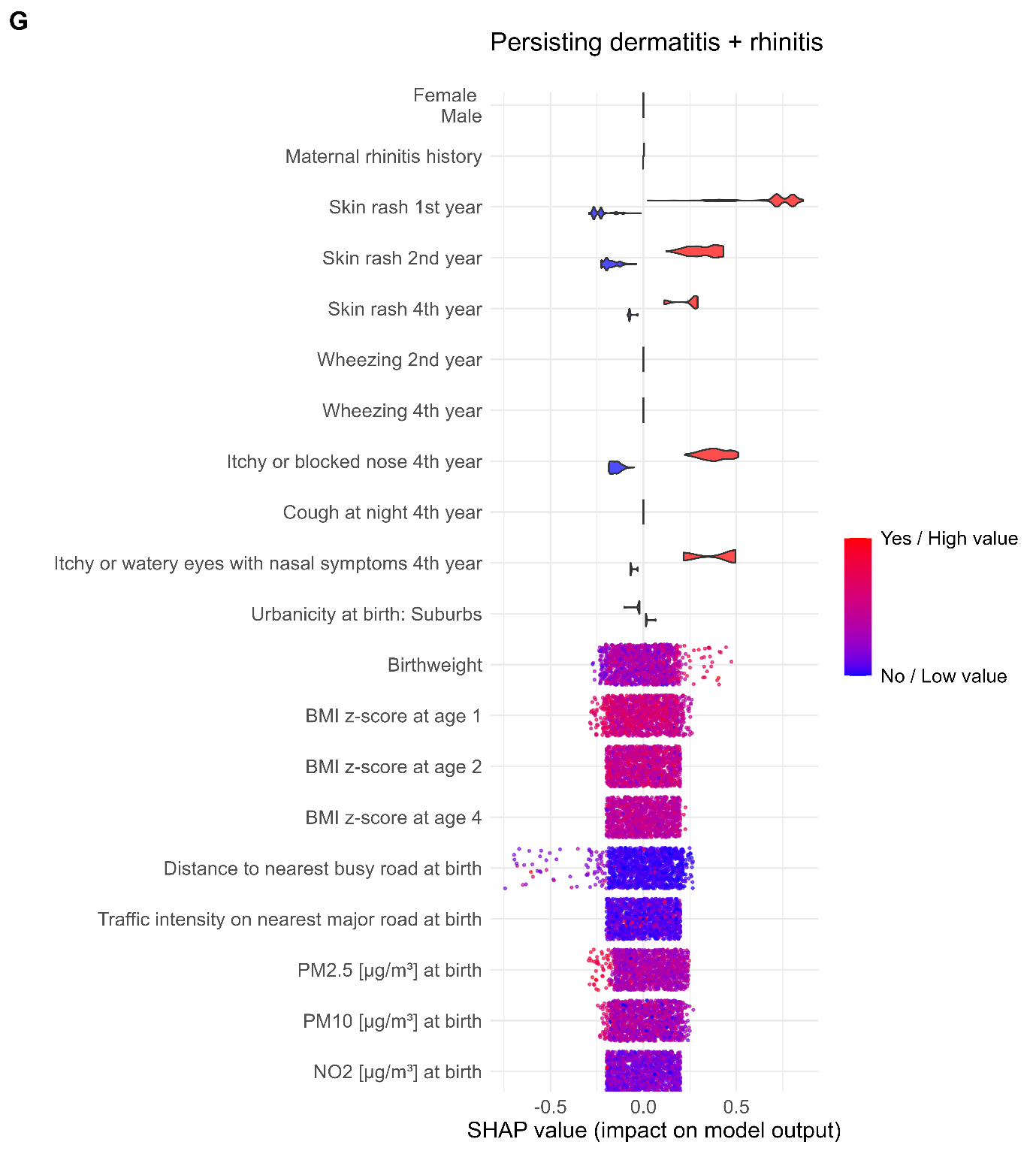


Figure S8: Receiver Operator Characteristic (ROC) curves per trajectory in training and test dataset, with PRS added to the predictor set, shown as mean performance complemented by 95% confidence intervals derived from performances across iterations over cross-validation folds and replicates for training and bootstraps for test data. Sample size per trajectory is shown for the training dataset followed by the values for the test dataset in brackets. Mean AUC values are presented for training and test data. Multiclass AUC training (test): 0.88 (0.64).


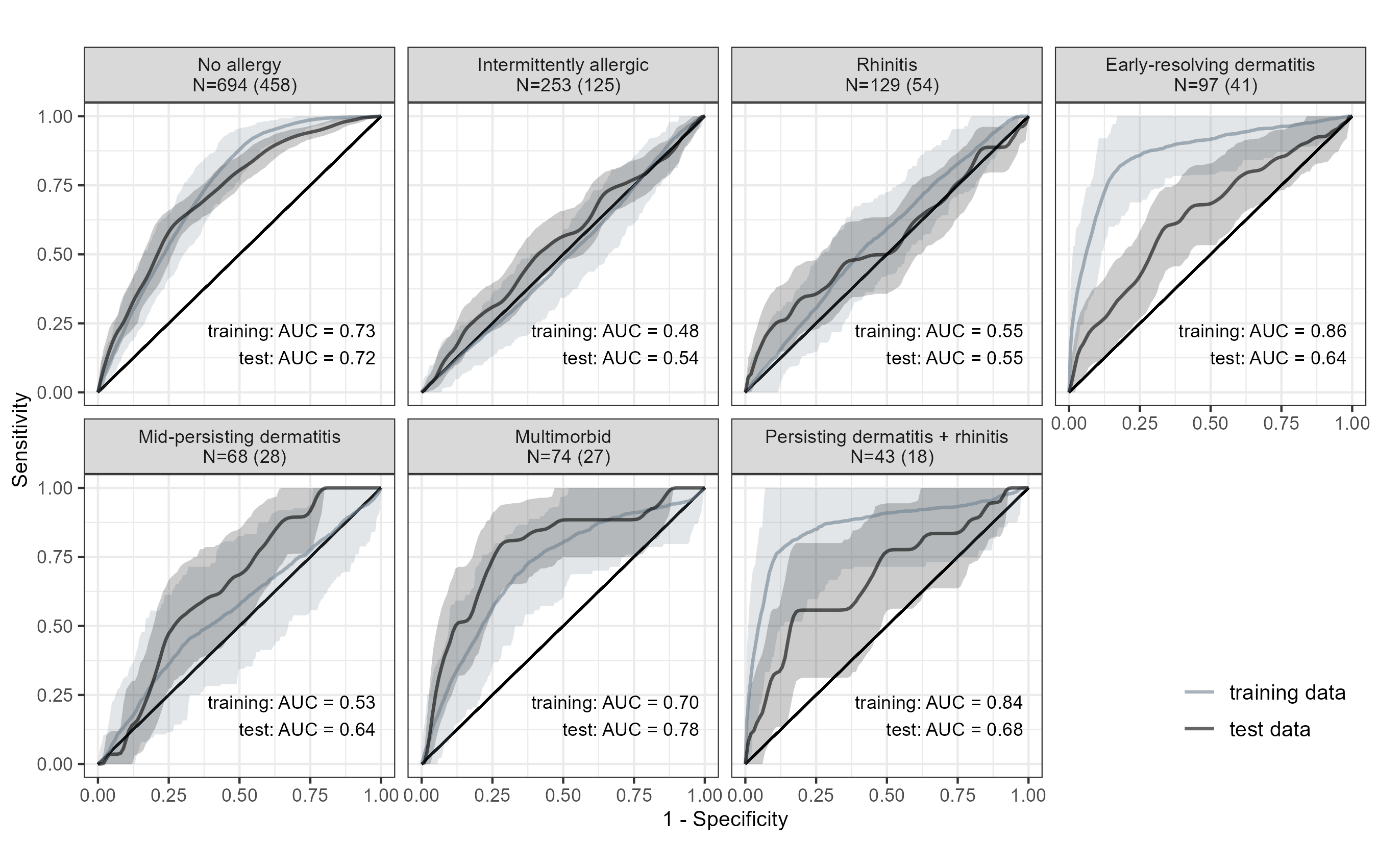


Figure S9: Receiver Operator Characteristic (ROC) curves per trajectory in training and test dataset when PRS were not included, shown as mean performance complemented by 95% confidence intervals derived from performances across iterations over cross-validation folds and replicates for training and bootstraps for test data. Sample size per trajectory is shown for the training dataset followed by the values for the test dataset in brackets. Mean AUC values are presented for training and test data. Multiclass AUC training (test): 0.86 (0.64).


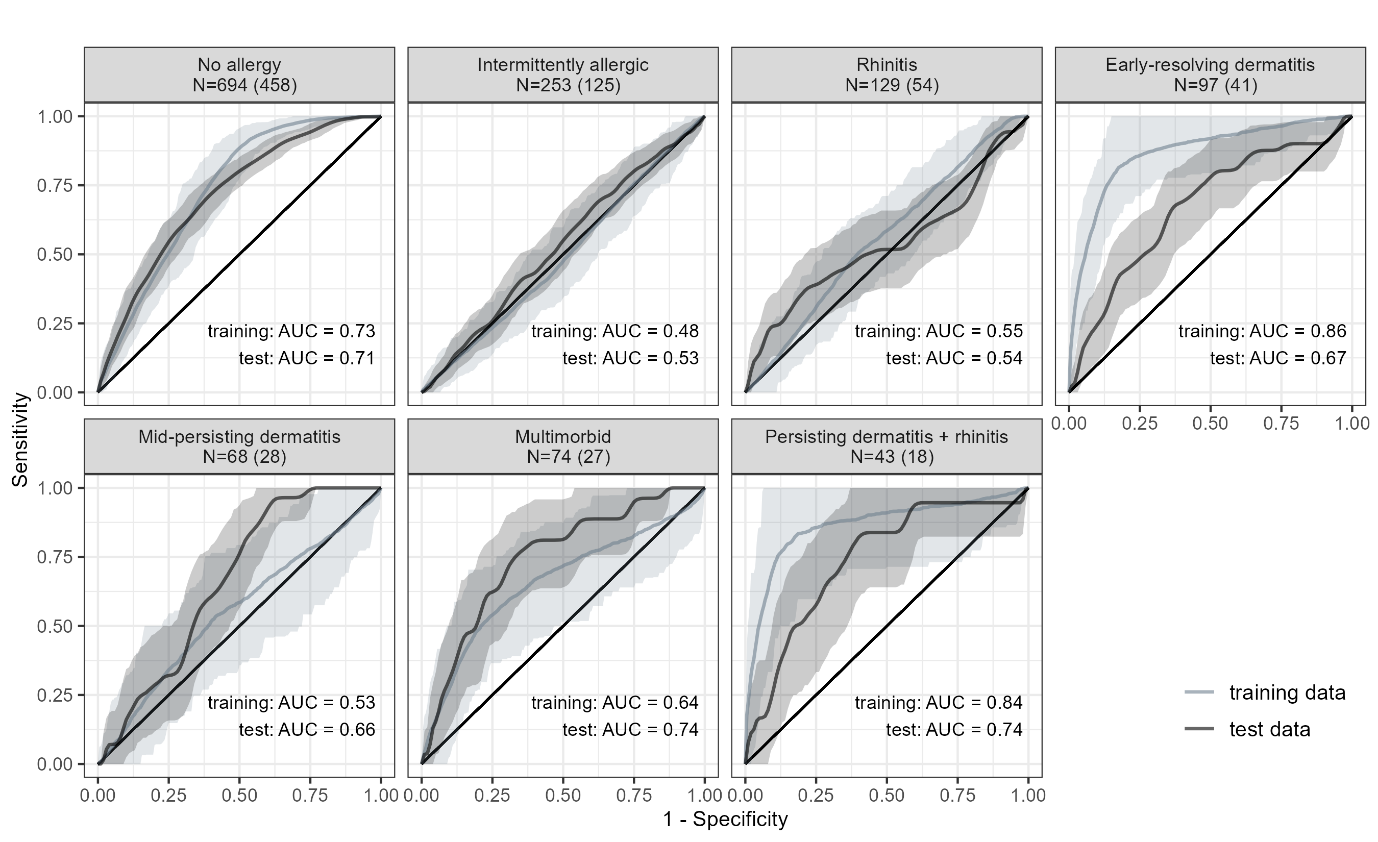


Figure S10: Variable importance for sub-analysis including polygenic risk scores, for all predictor variables, quantifying how useful each predictor is for improving the model’s overall prediction accuracy.


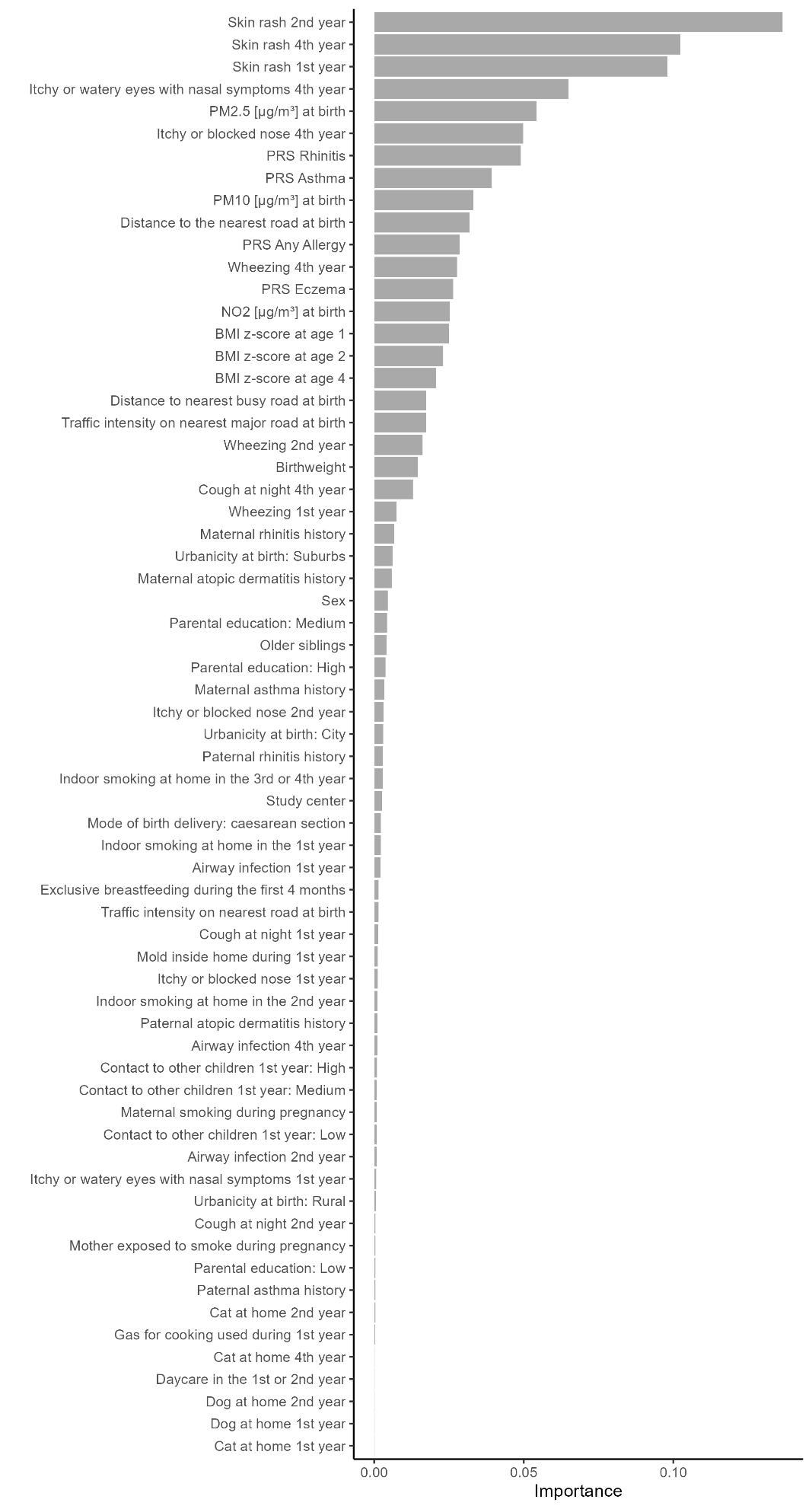


**References**

1. van Buuren S, Groothuis-Oudshoorn K. mice: Multivariate Imputation by Chained Equations in R. *Journal of Statistical Software* 2011; 45(3):1-67.

2. Chen T, Guestrin C: XGBoost: A Scalable Tree Boosting System. 2016.

3. XGBoost Explained: A Beginner’s Guide. Understand how XGBoost works, when to use it, and its advantages over other algorithms [<https://medium.com/low-code-for-advanced-data-science/xgboost-explained-a-beginners-guide-095464ad418f>]

4. McCarthy S, Das S, Kretzschmar W, Delaneau O, Wood AR *et al*. A reference panel of 64,976 haplotypes for genotype imputation. *Nat Genet* 2016; 48(10):1279-1283.

5. Das S, Forer L, Schönherr S, Sidore C, Locke AE *et al*. Next-generation genotype imputation service and methods. *Nature Genetics* 2016; 48(10):1284-1287.

6. El-Husseini ZW, Gosens R, Dekker F, Koppelman GH. The genetics of asthma and the promise of genomics-guided drug target discovery. *Lancet Respir Med* 2020; 8(10):1045-1056.

7. Waage J, Standl M, Curtin JA, Jessen LE, Thorsen J *et al*. Genome-wide association and HLA fine-mapping studies identify risk loci and genetic pathways underlying allergic rhinitis. *Nature Genetics* 2018; 50(8):1072-1080.

8. Budu-Aggrey A, Kilanowski A, Sobczyk MK, Shringarpure SS, Mitchell R *et al*. European and multi-ancestry genome-wide association meta-analysis of atopic dermatitis highlights importance of systemic immune regulation. *Nat Commun* 2023; 14(1):6172.

9. Ferreira MA, Vonk JM, Baurecht H, Marenholz I, Tian C *et al*. Shared genetic origin of asthma, hay fever and eczema elucidates allergic disease biology. *Nat Genet* 2017; 49(12):1752-1757.

10. Kilanowski A, Thiering E, Wang G, Kumar A, Kress S *et al*. Allergic disease trajectories up to adolescence: Characteristics, early-life, and genetic determinants. *Allergy* 2023; 78(3):836-850.

11. Kress S, Kilanowski A, Wigmann C, Zhao Q, Zhao T *et al*. Airway inflammation in adolescents and elderly women: Chronic air pollution exposure and polygenic susceptibility. *Science of The Total Environment* 2022; 841:156655.

12. Bloem M. The 2006 WHO child growth standards. *BMJ* 2007; 334(7596):705-706.
